# Metabolomic Insights into Marine Phytoplankton Diversity

**DOI:** 10.3390/md18020078

**Published:** 2020-01-25

**Authors:** Rémy Marcellin-Gros, Gwenaël Piganeau, Didier Stien

**Affiliations:** 1 Sorbonne Université, CNRS, Laboratoire de Biodiversité et Biotechnologie Microbiennes, LBBM, Observatoire Océanologique, 66650 Banyuls-sur-Mer, France; marcellin-gros@obs-banyuls.fr; 2 Sorbonne Université, CNRS, Biologie Intégrative des Organismes Marins, BIOM, Observatoire Océanologique, 66650 Banyuls-sur-Mer, France

**Keywords:** chemotaxonomy, phylogeny, mamiellales, galactolipids, betaine lipids, xanthophylls

## Abstract

The democratization of sequencing technologies fostered a leap in our knowledge of the diversity of marine phytoplanktonic microalgae, revealing many previously unknown species and lineages. The evolutionary history of the diversification of microalgae can be inferred from the analysis of their genome sequences. However, the link between the DNA sequence and the associated phenotype is notoriously difficult to assess, all the more so for marine phytoplanktonic microalgae for which the lab culture and, thus, biological experimentation is very tedious. Here, we explore the potential of a high-throughput untargeted metabolomic approach to explore the phenotypic–genotypic gap in 12 marine microalgae encompassing 1.2 billion years of evolution. We identified species- and lineage-specific metabolites. We also provide evidence of a very good correlation between the molecular divergence, inferred from the DNA sequences, and the metabolomic divergence, inferred from the complete metabolomic profiles. These results provide novel insights into the potential of chemotaxonomy in marine phytoplankton and support the hypothesis of a metabolomic clock, suggesting that DNA and metabolomic profiles co-evolve.

## 1. Introduction

Phytoplanktonic eukaryotes are phylogenetically highly diverse, as they have many representatives in most super-groups of the eukaryotic tree of life [1,2]. The Archaeplastida super-group, or green lineage [3], includes all of the species that have descended from a primary endosymbiosis event, when an ancestral eukaryotic cell engulfed a photosynthetic prokaryote, that eventually evolved into an organelle, the chloroplast [4]. Our knowledge on the diversity of phytoplanktonic green microalgae has greatly increased with the democratization of DNA sequencing and genomics, but it is likely to stay behind that of their terrestrial relatives, including land plants, whose estimated species number exceeds 400,000 [5]. This is not surprising since unicellular organisms are generally less studied than multicellular organisms, and because bona fide species identification relies on tedious sampling and isolation steps which have yet to be performed for many marine microalgae. Recently, DNA sequencing of numerous environmental marine water sample extracts collected worldwide during the Ocean Sampling Day initiative provided evidence that many of the sequences detected belong to species from the green lineage that have no representative strains in culture [6]. This study also demonstrated the very broad geographic distribution of species from the class Mamiellophyceae, that dominates the picoeukaryotic fraction (cell diameter <3 *µ*m) [7] in many coastal areas and thus plays a key ecological role in marine food webs. These picoalgae exemplify the ecological success of miniaturized eukaryotic cells [8] displaying a simple cellular organization (one mitochondrion and one chloroplast) and high surface–volume ratio, which is likely to confer advantages in nutrient-poor environments [9]. The Mamiellophyceae lineage is ancient, probably over 350 million years old [10], and currently comprises 22 described species [11]. Species from the three genera *Bathycoccus*, *Micromonas*, and *Ostreococcus* are particularly prevalent in the marine environment [6].

Historically, analysis of pigment composition has been used to assist the classification of microalgae. Indeed, *Bathycoccus*, *Micromonas*, and *Ostreococcus* species were found to contain characteristic pigments of many marine green microalgae, prasinoxanthin [12], but also specific pigments such as uriolide, micromonal, and dihydrolutein [13], which were not detected in other species outside the Mamiellales.

The advent of metabolomics now enables the metabolic signatures of microalgae to be explored at an unprecedented level of resolution [14]. These novel approaches have led to the discovery of new metabolites, fostered by the search for natural bioactive compounds with applications in agronomic, medical, or biofuel research. Indeed, polyunsaturated fatty acids (PUFA) are lipids with high nutritional value [15] and may have applications in the prevention of several pathologies, such as cancers or cardiovascular diseases [16,17]. Furthermore, the anti-inflammatory and antiviral properties of polar lipids have also been highlighted recently [18,19].

Here, we explored the potential of an untargeted metabolomic approach including pigments, lipids, and other uncharacterized metabolites to investigate chemotaxonomic markers in 12 marine microalgal strains from 11 species, including 9 microalgae from the green lineage; the Mamiellales *Ostreococcus tauri* [20], *O. mediterraneus* [21], *Bathycoccus prasinos* [22], *Micromonas commoda* [23], and *Mantoniella* sp., the Chlorellales *Picochlorum costavermella* [24], and strains from basal groups *Nephroselmis* sp. and *Pyramimonas* sp. To broaden the phylogenetic diversity of the dataset, two additional marine microalgae outside the green lineage were included: *Phaeodactylum tricornutum* (Stramenopile lineage) and *Pavlova lutheri* (Haptophyta lineage). We assess whether total metabolomic profiling enables us to delineate well-defined species, and we characterize the 10 major compounds of each species. Last but not least, we assess whether distances between metabolomic profiles, integrating both the composition and the frequency of each compound, reflect phylogenetic distances between species. This leads us to discuss the hypothesis of a metabolomic corollary of the molecular clock, a central tenet of molecular evolution.

## 2. Results

### 2.1. An Untargeted Holistic Analysis of Metabolomic Profiles

**Figure 1:**
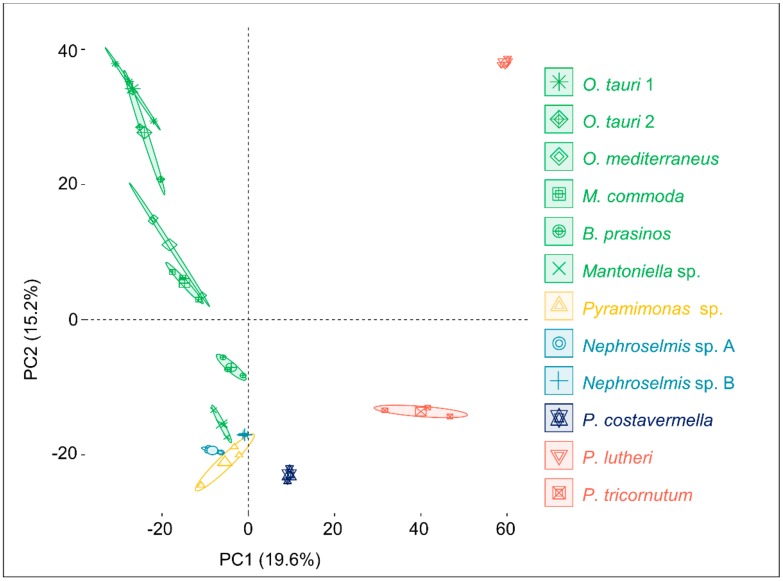
Principal Component Analysis of the whole metabolome of 12 marine microalgae. For each species and strain, confidence ellipses cover 95% of group position estimation.

To investigate metabolome diversity in 12 divergent algae, an untargeted holistic approach was chosen to cover a broad range of metabolites, such as lipids or pigments known as algal biomarkers. Algal ethyl acetate extracts were analyzed by UHPLC-ESI^+^-HRMS^2^, and acquired ion chromatograms were processed through an untargeted metabolomic workflow in Compound Discoverer 2.1 software (Thermo Scientific) to generate extracted ion chromatograms across samples, detect and quantify corresponding metabolites, and generate the observations/variables matrix used for further statistical analyses. As a first observation, 2565 ions were detected across all samples, and 1143 ions were unique features detected in only one species. Moreover, detected features ranged from 867 ions in *O. tauri* sp. 1 to 241 ions in *Pyramimonas* sp. 1422 ions are shared between at least two species (Figure S1). These observations suggest high variation in the diversity of produced metabolites. To display this variation, an initial principal component analysis (PCA) was conducted on the observations/variables matrix to compare metabolomic profiles between strains at the global metabolome scale (Figure 1). Principal Components (PCs) 1 and 2 describe 34.8% of the variation, and the first PC separates Mamiellales from the two microalgae *P. lutheri* (Haptophyta lineage) and *P. tricornutum* (Stramenopile lineage), hereafter considered outlier microalgae as they are not part of the green lineage. The second PC separates *Ostreococcus* species from other Chlorophytes (*Nephroselmis*, *Pyramimonas*). Interestingly, intraspecific metabolome diversity seems minimal as compared to within-replicate variation, as the two *O. tauri* strains’ PCA confidence ellipses overlap.

### 2.2. Identification of the Major Metabolites and Detection of Chemotaxonomic Markers

**Figure 2:**
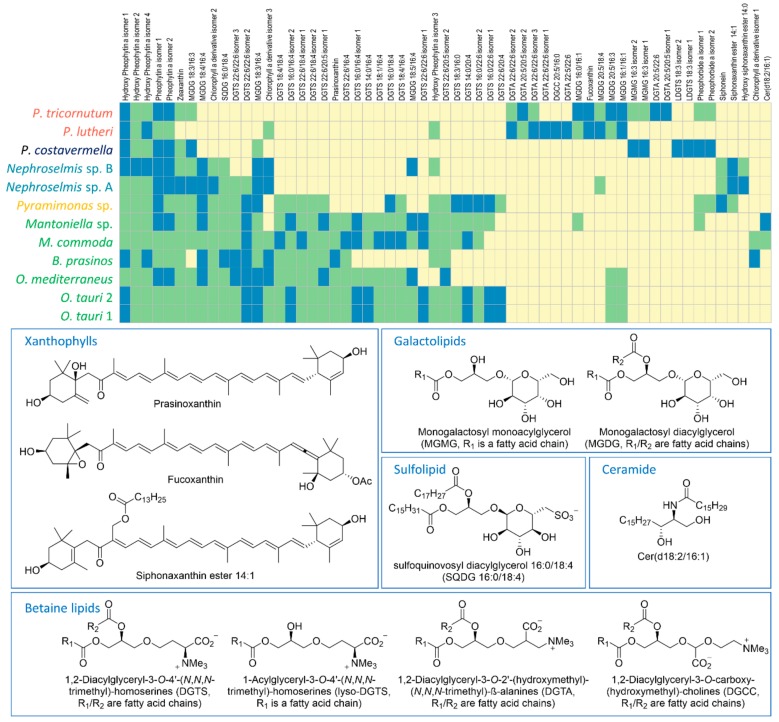
Matrix of the top 10 (blue), detected (green), and not detected (yellow) metabolites, and structure examples of the 59 most abundant compounds over the 12 microalgal strains. For all compounds, identifications are based on (1) molecular formulas, (2) automatic assignment via Global Natural Products Social Molecular Networking (GNPS), (3) interpretation of MS^2^ spectra and comparison with published data, and (4) phylogeny.

In order to go further into the comparative metabolic profiling of the 12 strains, the 10 major metabolites of each strain, defined as the 10 highest peak areas of the extracted ion chromatograms (XIC), were identified (Figure 2). Identification was carried out by comparing compound raw formulas (calculated on the basis of high-resolution mass spectrometry) to databases (Dictionary of Marine Natural Products and SciFinder) to retrieve candidate compounds, then MS^2^ spectra were submitted to databases for comparison (Global Natural Products Social Molecular Networking—GNPS [25]) or elucidated to infer putative structures. Compounds were classified into 10 different groups of polar lipids and pigments. Among the polar galactolipids, eight were monogalactosyl diacylglycerols (MGDGs) (Figure S2), two were monogalactosyl monoacylglycerols (MGMGs or Lyso-MGDGs), and one was a sulfoquinovosyl diacylglycerol (SQDG) (Figures S4 and S5).

For MGDGs and SQDGs, the regiochemical assignment (*sn*-1 and *sn*-2 positions) of both fatty acid (FA) chains was done by comparing the MS^2^ fragmentation patterns. The fragment resulting from *sn*-1 FA loss ([M+X-R_1_CO_2_H]^+^) exhibits a higher peak intensity than the one resulting from *sn*-2 FA loss ([M+X-R_2_CO_2_H]^+^) for the protonated adduct (X = H) of SQDGs [26] and sodiated adduct (X = Na) of MGDGs [27]. Besides this, it has been established in the literature that the sugar moiety is a galactose for glycolipids and a sulfoquinovose for SQDG [28]. The betaine lipids are the most represented and diversified group with twenty 1,2-diacylglyceryl-3-*O*-4′-(*N*,*N*,*N*-trimethyl)-homoserines (DGTSs) (Figures S6 and S7), seven 1,2-diacylglyceryl-3-*O*-2′-(hydroxymethyl)-(*N*,*N*,*N*-trimethyl)-ß-alanines (DGTAs), two 1-acylglyceryl-3-*O*-4′-(*N*,*N*,*N*-trimethyl)-homoserines (lyso-DGTSs), and one 1,2-diacylglyceryl-3-*O*-carboxy-(hydroxymethyl)-choline (DGCC) (Figure S8). Regiochemical assignment of FAs was done as for galactolipids on the basis of MS^2^ spectra. Here, the collision-induced [M + Na-R_2_CO_2_H]^+^ fragment of sodiated adducts produced a higher peak than the [M+Na-R_1_CO_2_H]^+^ fragment [29]. Distinction of the isomeric DGTA and DGTS betaine lipids was performed on the basis of strain phylogeny in case of coelution. The fragmentation pattern commonly described for these lipids includes the characteristic 59 Da neutral loss corresponding to the loss of trimethyl amine (NMe_3_) and the 87 Da neutral loss (CH_3_-CH^-^-N^+^Me_3_) for DGTSs as a consequence of fragmentation after transposition of the carboxyl group [30]. Unfortunately, this fragmentation reaction was not observed in our analyses. According to the literature, DGTAs are specific to brown algae (*P. lutheri* and *P. tricornutum*) while DGTSs are produced by microalgae from the green lineage [28]. On the pigment side, 11 chlorophylls (Figures S9–S23) and 6 xanthophylls (Figures S24–S30) appeared to be largely shared among strains. In the xanthophyll series, one compound was identified as either prasinoxanthin or its isomer violaxanthin. The uncertainty was eventually disentangled thanks to the typical dehydration fragmentation pattern of prasinoxanthin (Figure S28), while an 80 Da neutral loss was observed for the epoxycarotenoid violaxanthin (Figure S29) [31]. Fucoxanthin (Figure S30) was unambiguously identified from specific fragments at *m*/*z* 109.1014, 581.3975, and 641.4207 in MS^2^ [32]. Apart from the polar lipids and pigments, a ceramide non-hydroxy fatty acid sphingosine (Cer) (Figure S31) was also identified.

The most abundant and diversified metabolites identified over the 12 strains were polar lipids and pigments. These observations are consistent with an increasing number of studies concerning the analysis of algal lipidomes [28,33] and provide new lipidome information for the strains *Mantoniella* sp., *Nephroselmis* sp., and *Pyramimonas* sp. recently isolated from environmental samples. Further phylogenetic signals of metabolites are given and discussed in the following section.

**Figure 3:**
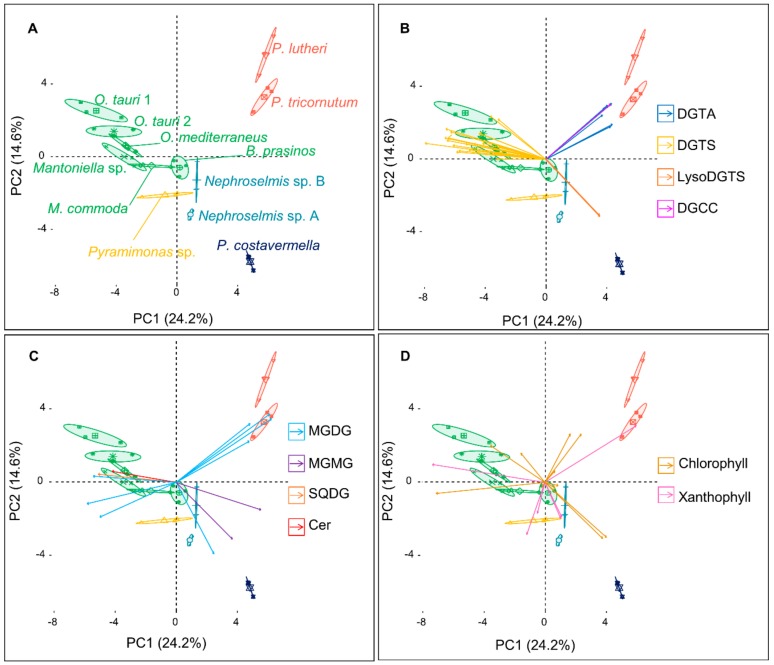
(**A**) Principal component analysis (PCA) constructed from the 59 most abundant metabolites matrix over the 12 microalgal strains, and the corresponding biplots of the (**B**) betaine lipids, (**C**) galacto-, sulfolipids, and ceramide, and (**D**) pigments (each arrow corresponds to a metabolite). Confidence ellipses cover 95% of group position estimation. Arrow coordinates correspond to the contributions of metabolites to the PC and color to the metabolite class. Arrows point toward the strains where they are the most represented.

The 10 major metabolites of each microalga shown above were chosen to construct a new matrix of compound abundance to perform a second PCA (Figure 3A). Remarkably, the variability explained by the two PCs remained similar (38.8%), and so did the pattern of clustering of the different strains as compared to the first PCA conducted on the whole metabolome analysis. The first PC again discriminated the outlier microalgae *P. lutheri* and *P. tricornutum* from the Mamiellales, while PC2 separated *P. costavermella* from the brown algae, and the green microalgae fanned out along this axis.

The contribution of each major metabolite to a strain or group of algae can be inferred from the biplot projection of the PCA (Figure 3B–D). The first PC separates the green microalgae from the outlier brown ones, and as expected, this distinction is primarily due to the betaine lipids DGTAs and DGTSs. The high chemical diversity of DGTSs is due to a greater variability in the acyl chain length and number of unsaturations, while DGTAs hold only long (C20–C22) and highly unsaturated acyl chains (Figure 2). MGDGs are represented in every species. MGDGs 18:3/16:4, 18:4/16:4, and 18:5/16:4 are only found in green microalgae. They predominate in the Mamiellales as previously described by Degraeve-Guilbault et al. [34]. Major galactolipids of the brown algae *P. tricornutum* contain 16:0, 16:1, 16:3, and 20:5 fatty acid chains, which is also consistent with previous analyses, reinforcing the reproducibility of these observations [29]. Interestingly, MGMGs are only detected in *P. tricornutum* and *P. costavermella*. Usually, these lipids are not extensively studied in the literature and may be associated with lipid remodeling or environmental plasticity [35]. Some strains can exhibit metabolites exclusive to their group. DGCCs are present in *P. lutheri* but absent in *P. tricornutum* and may be a biomarker of haptophytes [28,36]. This is largely described in the literature, but we also show here that fucoxanthin occurs in the lineage containing *P. lutheri* and *P. tricornutum*. On the other hand, C14 esterified siphonaxanthin (Figures S25–S27) and siphonein (Figure S29) are specific to the Chlorophytes *Nephroselmis* and *Pyramimonas*. In fact, these pigments are widely found in green algae, especially in deep-water or shade species, as they improve the efficiency of light-harvesting complexes [37] or protect the cells from high light damage. Moreover, prasinoxanthin (Figure S31) is the major xanthophyll that identifies Mamiellales amongst other green algae [12].

Beyond a simple identification of metabolite classes within microalgal species or lineages, our results demonstrate that some specific metabolites within classes may serve as phylogenetic markers, pending analysis of additional species identified in each group. Our analysis confirms that prasinoxanthin is specific to the Mamiellales, but also that DGTS 22:6/16:4 is specific to microalgae from this group. The number and diversity of DGTSs we found between *Pyramimonas* sp. and Mamiellales is an outstanding observation, since these betaine lipids were not detected in *Nephroselmis* sp. or even in *B. prasinos* or *O. mediterraneus*. Within the Mamiellales, the *Ostreococcus* genus differs by the presence of two MGDGs (20:5/16:3 and 16:1/16:1). The species *O. tauri* has six DGTSs which are not found in *O. mediterraneus*. Interestingly, the presence/absence matrix does not differentiate both *O. tauri* strains. More data will be necessary to confirm these observations, but the “major metabolites approach” does indeed appear to be interesting for differentiating between these microalgae at the species level.

The choice of taxonomic chemical biomarkers is always challenging as the diversity and abundance of metabolites should reflect species divergence rather than intrinsic variability due to environmental factors. To get around this, chemical classification of plants has been preferentially achieved by comparison of secondary metabolites since these are remarkably diverse [38], include numerous classes of compounds (glycosides, phenolics, or alkaloids) [39], and are relevant for species classification, as they are restricted to taxonomically related groups of species [40,41]. Comparison of algae has so far relied on pigment analysis using 44 pigment types spanning 27 classes of photosynthetic algae [42], and these pigments are consistent with the endosymbiotic evolutionary history of eukaryotes [43]. More recently, many efforts in algal compound screening have enabled the description of hundreds of new metabolites each year [44], which provide the opportunity to identify species from a broader spectrum of compounds. Algal lipids have been extensively described in model species such as *Chlamydomonas reinhardtii*, *Chlorella* sp., *Nannochloropsis* sp., or *P. tricornutum* [29,45,46,47], while complete lipidome profiles have yet to be acquired for most algae. Nonetheless, lipids, especially FAs [48,49,50], sterols [50], alkenones [51], or polar lipids [52,53], are widely used as species tracers. It is important to keep in mind that lipid profiles may be impacted by environmental, biotic, or abiotic factors [54], as demonstrated in many studies on nutrient availability, irradiance, and growth stage [29,34,55,56]. However, even though taxonomic signals may be diminished by external factors, it has been shown that taxonomy accounts for 3 to 4 times more variance in the lipid profiles of phytoplankton than abiotic factors [49]. Besides this, polar lipids, especially betaine lipids (DGTA/S, DGCC), constitute the least impacted metabolite class by growth stage as demonstrated by Cañavate and colleagues, and they are therefore considered reliable lipidic taxonomic markers [57]. The abundancy profiles of “major metabolites” are consistent across experiments from available studies performed at the molecular level [29,34,58], suggesting that they are relevant chemotaxonomic marker candidates. 

### 2.3. Phylogenetic Analysis and Metabolome-Based Taxonomy 

**Figure 4:**
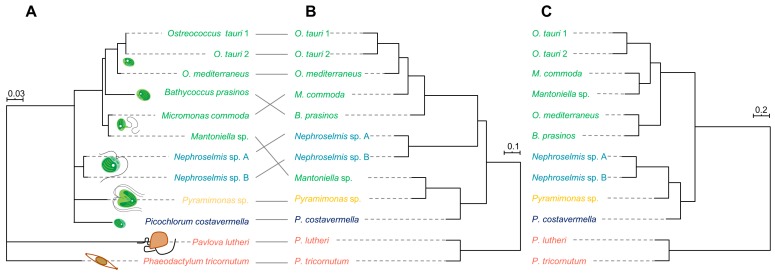
(**A**) Partial 18S ribosomal gene phylogeny based on 429 base pairs; scale indicates substitution per site. (**B**) A hierarchical clustering dendrogram based on 3138 metabolites; the scale represents the Spearman correlation coefficient between strains. (**C**) A hierarchical clustering dendrogram based on the 59 most abundant metabolites; the scale represents the Spearman correlation coefficient between strains.

The phylogeny based on the partial analysis of the 18S rDNA subunit was consistent with previous findings based on a larger set of sequence data [1,59] retracing the molecular divergence between species and the different microalgal families (Figure 4A) and was therefore used as a reference for comparison with the information from the metabolome of each microalga.

First, hierarchical clustering analysis (HCA) was performed to determine the metabolomic proximity between strains. The HCA dendrogram (Figure 4B) was generated by calculating the distance matrix between strains based on the Spearman correlation coefficient and aggregated by “complete linkage”. A clear clustering of Mamiellales species emerged from HCA, except for *Mantoniella* sp., which exhibited a metabolomic profile more closely related to *Pyramimonas* sp. This peculiarity disappears when the metabolome is reduced to the 10 major metabolites to build the chemotaxonomy (Figure 4C). This suggests that major compounds tend to be reliable species biomarkers when comparing divergent as well as closely related organisms. Microalgal cultures are very rarely axenic, since a bacterial community often co-exists with microalgae [60]. Estimation of the proportion of bacteria by cytometry revealed that most cultures contained less than 5% bacteria, while it turned out that both *Mantoniella* sp. and *Pyramimonas* sp. could not be cleansed of bacterial partners (microalgae/bacteria ~1:1). This may be a consequence of their mixotrophic regime [61,62]. The clustering of these strains may thus be due to the metabolomic contribution of bacteria, either because of identical metabolites of bacterial origin or through a similar microalgae–bacteria interaction process that may dramatically influence the metabolome of the microalgal partner. However, we demonstrate here that algal metabolites predominate in the total extract, so bacterial participation disappears when considering the major metabolites only. Besides this, statistical comparison of genetic distances with metabolomic distances based on whole-metabolome analysis amongst algae confirms a strong correlation between both approaches (Mantel test: *r* = 0.77, *p*-value = 0.001). Thus, there is a good overlap of phylogenetic and chemotaxonomic signals, as metabolomic distances reflect genetic distances between species. This correlation is even stronger when estimated from the most abundant metabolites (Mantel test: *r* = 0.90, *p*-value = 0.001), confirming the robustness and potential of the metabolomic approach to discriminate and retrace the evolutionary history of divergent species.

Establishing the full lipidome profile of algae at the molecular level within and between species is relevant for several reasons. First, it provides a fundamental description of the species at the metabolite level and helps to estimate the chemical divergence between strains or species. Secondly, it can lead to the identification of algal species producing interesting bioactive compounds, such as high-value carotenoids and lipids, with agronomical and pharmaceutical applications. The present study highlights the abundance of long-chain polyunsaturated FA, such as octadecapentaenoic acid (18:5n-3), eicosapentaenoic acid (20:5n-3), or docosahexaenoic acid (22:6n-3), the health benefits of which have been previously recognized [63], for example, in preventing cardiovascular or mental disorders [64,65,66]. Moreover, particularly abundant in Mamiellophyceae, polar lipids and especially MGDG 18:3/16:4 and MGDG 18:4/16:4 of *Tetraselmis* sp. have effective anti-inflammatory properties [67,68]. *P. lutheri*’s major galactolipid MGDG 20:5/18:4 has also been shown to have anti-inflammatory activity [69], inhibiting both human melanoma cell growth [70] and bacterial development [71]. Last but not least, SQDGs are probably among the most promising compounds in the medical field as lipids from this class are known to act on HIV infection [72], but also exhibit anti-HSV-1 and anti-HSV-2 activities [73] and anti-inflammatory [74] and antitumor properties [75]. The aforementioned bioactive molecules are far from exhaustive but highlight the biotechnological potential of algae as producers of bioactive molecules. 

In conclusion, we investigated the metabolomes of 12 microalgal strains from 11 species and characterized the major carotenoids and lipids. The approach of using major metabolites allows microalgal species and lineages to be distinguished. Evolutionary divergence between species can be inferred, in good congruence with the phylogenies obtained from sequence data obtained through a classical molecular approach. Therefore, these results support the hypothesis of a metabolomics equivalent to the “molecular clock” based on the analysis of sequence data. The resulting “metabolomics clock” metaphor is also constrained by the technical challenges raised by the “molecular clock” inferred from DNA sequence analysis: What are the mode and tempo of metabolome evolution? Are some metabolites changing faster than others? What is the distribution of fitness effects of metabolite changes? To answer these questions, statistical developments are needed to develop metabolomic distances and larger datasets including additional species should be obtained to include a broader variation in evolutionary distances.

## 3. Materials and Methods

### 3.1. Culture Conditions and Growth Measurement

Cultures were grown in modified Keller Artificial Seawater medium [76] (K-ASWO) containing 420 mM NaCl, 10 mM KCl, 20 mM MgCl_2_, 10 mM CaCl_2_, 25 mM MgSO_4_, 2.5 mM NaHCO_3_, 0.88 mM NaNO_3_, 5.0 × 10^−5^ M NH_4_Cl, 1.0 × 10^−5^ M β-glycerophosphate, 1.0 × 10^−8^ M H_2_SeO_3_, 1 mL of 1 M Tris-HCl (pH 7.2) per liter of medium, 3.7 × 10^−10^ M cyanocobalamin, 2.0 × 10^−9^ M biotin, and 3.0 × 10^−7^ thiamine in addition to Keller trace metal solution [77]. Algal strains were cultured in T75 cell culture flasks with ventilated caps (Sarstedt, Germany) containing 100 mL of K-ASWO medium. Each flask was inoculated to a cell density of 1 × 10^6^ cells.mL^-1^ for *O. tauri* 1 (RCC 6850) and 2 (RCC 4221), *O. mediterraneus* (RCC 2590), *B. prasinos* (RCC 4222), *M. commoda* (RCC 827), *Mantoniella* (RCC 6849), *P. costavermella* (RCC 4223), *P. tricornutum* (RCC 6851), and *P. lutheri* (RCC 6852); 2.4 × 10^5^ cells.mL^−1^ for *Nephroselmis* sp. A (RCC 6846); 3.3 × 10^5^ cells.mL^−1^ for *Nephroselmis* sp. B (RCC 6847); and 7.8 × 10^4^ cells.mL^−1^ for *Pyramimonas* sp. (RCC 6848). Cultures were maintained at a temperature of 20 °C under continuous light of 100 µE.m^-2^.s^-1^ and were agitated manually once a day; cell density was measured every day by flow cytometry.

### 3.2. Microalgal Culture Axenization

All cultures were treated with antibiotics to lower the bacterial concentration. Quantities of 50 µg.mL^−1^ ampicillin (A9518, Sigma-Aldrich), 50 µg.mL^−1^ gentamycin (G1914, Sigma-Aldrich), 20 µg.mL^−1^ kanamycin (60615, Sigma-Aldrich), and 100 µg.mL^−1^ neomycin (N6386, Sigma-Aldrich) were added to K-ASWO, and after two subculturing stages, the bacterial content was low enough to perform metabolomic analysis in most strains. A single antibiotic treatment reduced the bacterial contamination in *Ostreococcus* and *P. lutheri* cultures but was unable to remove bacteria completely in others.

### 3.3. Flow Cytometry

Cells were fixed using glutaraldehyde (0.25% final concentration, G6257, Sigma-Aldrich) with the addition of Pluronic F-68 (0.1% final concentration, P-7061, Sigma-Aldrich) for 15 min in the dark and stained with SYBR Green I (LON50512, Ozyme) for another 15 min in the dark. Cell counting was performed using a Beckman Coulter Cytoflex flow cytometer (laser excitation wavelength 488 nm) by chlorophyll autofluorescence for microalgae (detection filter >620 nm) and by SYBR Green I fluorescence for bacteria (detection bandwidth 525–540 nm, corresponding to the FITC (fluorescein isothiocyanate) channel). Data were analyzed with CytExpert 2.2 software (Beckman Coulter).

### 3.4. Metabolite Extraction

Microalgal cells were collected three to four days post inoculation (Supplemental Figure S1) by filtration of 100 mL of culture through a Whatman GF/F filter (Z242519, Sigma-Aldrich) under reduced pressure (600 mbar). Then, filters were placed in disposable glass culture tubes with 7 mL of ethyl acetate (16371, Sigma-Aldrich) to solubilize algae cells overnight in a C25 incubator shaker (New Brunswick Scientific, 100 rpm, 19 °C).

### 3.5. UHPLC-HRMS Analyses

Microalgal extracts were analyzed on an Ultimate 3000 UHPLC Dionex system coupled to an Orbitrap MS/MS FT Q-Exactive Focus Thermo Scientific mass spectrometer. Samples were solubilized in MeOH (1 mg.mL^−1^) and 1 µL was injected onto the column. The column was a Phenomenex Luna Omega Polar C18 (150 × 2.1 mm, 1.6 µm, 100 Å) conditioned at 42 °C. The mobile phase was a mixture of water (solvent A) with increasing proportion of acetonitrile (solvent B, 012041, Biosolve), both solvents modified with 0.1% of formic acid. The gradient was as follows: 50% B from 3 min before injection to 1 min after; between 1 and 3 min, a linear increase of B up to 85%, followed by 85% B for 2 min; 89% B from 5.1 to 7 min; 93% B from 7.1 to 10 min; 97% B from 10.1 to 13 min; and finally, 100% B from 13.1 to 18 min. The flow was set to 0.5 mL.min^−1^ and injected into the mass spectrometer 1 min after injection (diverted before). Mass spectrometry analyses were performed in the positive electrospray ionization mode in the 133.4–2000 Da range, and mass spectra were recorded in the centroid mode. The mass spectrometer method was set to FullMS data-dependent MS^2^. In fullMS, the resolution was set to 70,000 and the AGC target to 3 × 10^6^ for a chromatogram peak width (FWHM) of 6 s. In MS^2^, the resolution was 17,500, the AGC target 1 × 10^5^, the isolation window 0.4 Da, and the stepped normalized collision energy 15/30/45 with 10 s of dynamic exclusion. The lock mass was calibrated on the Cu(CH_3_CN)^2+^ ion at *m/z* 144.9821 Da.

### 3.6. LC-MS Data Preprocessing

Total ion chromatograms were processed through the untargeted metabolomic workflow of Compound Discoverer (CD) 2.1 (Thermo Scientific). A Quality Control mix (QC) composed of the 12 algal extracts was analyzed together with algal extracts and K-ASWO medium used as a blank to remove nonalgal compounds. The CD workflow performs retention time correction, detection of unknown compounds, and grouping across samples; fills gaps when features are absent; hides chemical background (using blank samples); and finally predicts compound elemental composition. The retention time window was set to 2–18 min. The maximum time shift for compound alignment was 0.1 min, the maximum mass tolerance for compound grouping and elemental composition calculation was 3 ppm, and the minimum peak intensity was 2 × 10^6^. This workflow provided an observation/variable matrix used for further statistical analysis. 

### 3.7. Confirmation of Algae Identities and Reconstruction of Phylogenies

Algae identification was performed on the basis of partial 18S rDNA sequence analysis. Total DNA was extracted with hexadecyltrimethylammonium bromide (CTAB) as described by Winnepenninckx et al. [78]. The 18S rDNA gene region was amplified by PCR using the unique nondegenerate universal eukaryotic forward primer F-566 (5’ CAG CAG CCG CGG TAA TTC C 3’) and the reverse primer R-1200 (3’ CCC GTG TTG AGT CAA ATT AAG C 5’) [79] before sequencing by the GATC company.

Then, partial 18S rDNA sequences were aligned using MUSCLE 3.8 [80], and gaps were manually removed to get 429 base pairs sequences. Best substitution model selection and phylogenetic tree reconstruction was performed using IQ-TREE 1.6.12. The best selected model was TIM2e+G4 with the Bayesian Inference Criterion, and 1000 bootstraps were used to construct the consensus phylogenetic tree.

### 3.8. Figure Plotting and Statistical Analysis

All figures were plotted using R 3.6.1. Principal Component Analysis and corresponding biplots were calculated and constructed using the FactoMineR v1.42 package and PCA function with the scaled data option and 95% confidence ellipse lines. The phylogenetic tree was plotted using the phytool v0.6-99 package, and the patristic matrix was calculated using the ape v5.3 package, while the metabolite distance correlation matrix was calculated using the R base stats functions. The Mantel test was performed using the ade4 v1.7-4 package.

## Supplementary Materials

The following are available online at https://www.mdpi.com/1660-3397/18/2/78/s1, Figure S1: Histogram of detected compounds (A) and occurrence in algal strains (B), Figure S2: Microalgal concentration growth curves over time, Table S1: Algal and bacterial concentration (cells.mL^−1^) at sampling day for the 12 algae species, Figures S3–S31: Analytical data on microalgal metabolites.
