# Supplementary material for: Metabolomic Insights into Marine Phytoplankton Diversity
Source: Mar Drugs. 2020 Jan 25;18(2):78. doi: 10.3390/md18020078 (PMC7074452; doi:10.3390/md18020078)
Supplement: Supplementary file 1 [file marinedrugs-18-00078-s001.pdf]

Supplementary material for

Metabolomic insights into marine phytoplankton diversity

Rémy Marcellin-Gros [1,2], Gwenaël Piganeau [2,*] and Didier Stien [1,*]

[1] Sorbonne Université, CNRS, Laboratoire de Biodiversité et Biotechnologie Microbiennes, LBBM, Observatoire Océanologique, 66650 Banyuls-sur-Mer, France
[2] Sorbonne Université, CNRS, Biologie Intégrative des Organismes Marins, BIOM, Observatoire Océanologique, 66650 Banyuls-sur-Mer, France
* Correspondence: gwenael.piganeau@obs-banyuls.fr; Tel.: +33-468887343 (G.P.). didier.stien@cnrs.fr; Tel.: +33-430192476 (D.S.)

Table of Content

Figure S1. Stacked histogram of detected compounds (bottom bars, intense colors) among total detected compounds (upper bars, blurred colors) in the twelve strains of algae (**A**). Histogram of counts of shared compounds between different strains (abscise axis indicates the number of strains sharing the same compound). Most of compounds (1143) are uniquely detected in one strain (1) while only few compounds (18) are detected in every alga (12) (**B**). 3

Figure S2. Microalgae concentration growth curves over time. Yellow arrows: metabolite extraction was performed at 3 days post-inoculation for Ostreococcus species and 4 days post inoculation for the others. 3

Table S1. Algal and bacterial concentration (cells.ml^{-1}) at sampling day for the twelve algae species. RCC for Roscoff Culture Collection numbers for strain identification. 4

Figure S3. Compound M3629 – *m/z* 793.4874 – [M+Na]$^+$ – t_R = 8.976 min – $C_{45}H_{70}O_{10}$ – MGDG 20:5/16:3 4

Figure S4. Compound M5273 – *m/z* 815.4985 – [M+H]$^+$ – t_R = 9.167 min – $C_{43}H_{74}SO_{12}$ – SQDG 16:0/18:4 4

Figure S5. Fragmentation pathway proposed for SQDGs 5

Figure S6. Compound M2531 – *m/z* 804.578 – [M+H]$^+$ – t_R = 8.976 min – $C_{50}H_{77}NO_7$ – DGTS 22:6/18:4 5

Figure S7. Fragmentation pathway proposed for DGTSs (Fragmentation pattern of the DGTAs are similar) 5

Figure S8. Compound M3834 – *m/z* 774.5888 – [M+H]$^+$ – t_R = 9.167 min – $C_{46}H_{79}NO_8$ – DGCC 16:0/20:5 6

Figure S9. Compound M6284 – *m/z* 932.4940 – [M]$^+$ – t_R = 13.397 min – $C_{56}H_{68}MgN_4O_7$ – Chlorophyll a derivative 1 6

Figure S10. Compound M6982 – *m/z* 952.5200 – [M]$^+$ – t_R = 15.072 min – $C_{56}H_{72}MgN_4O_8$ – Chlorophyll a derivative 2 7

Figure S11. Compound M7441 – *m/z* 938.5412 – [M]$^+$ – t_R = 16.286 min – $C_{56}H_{74}MgN_4O_7$ – Chlorophyll a derivative 3 7

Figure S12. Proposed fragmentation pathway for Compound M7441 7

Figure S13. Compound 7354 – *m/z* 871.5740 – [M+H]$^+$ – t_R = 16.096 min – $C_{55}H_{74}N_4O_5$ – Pheophytin a isomer 1 8

Figure S14. Proposed fragmentation pathway for Compound M7354 8

Figure S15. Compound 7377 – *m/z* 871.5740 – [M+H]+ – t_R = 16.169 min – $C_{55}H_{74}N_4O_5$ – Pheophytin a isomer 2 9

Figure S16. Compound M5963 – *m/z* 887.5688 – [M+H]+ – t_R = 12.658 min – $C_{55}H_{74}N_4O_6$ – Hydroxy-pheophytin isomer 1 9

Figure S17. Proposed fragmentation pathway for Compound M5963 9

Figure S18. Compound M6021 – *m/z* 887.5688 – [M+H]+ – t_R = 12.779 min – $C_{55}H_{74}N_4O_6$ – Hydroxy-pheophytin isomer 2 10

Figure S19. Compound M6695 – *m/z* 887.5688 – [M+H]+ – t_R = 14.279 min – $C_{55}H_{74}N_4O_6$ – Hydroxy-pheophytin isomer 3 10

Figure S20. Compound M6726 – *m/z* 887.5688 – [M+H]+ – t_R = 14.396 min – $C_{55}H_{74}N_4O_6$ – Hydroxy-pheophytin isomer 4 11

Figure S21. Compound M2385 – *m/z* 593.2763 – [M+H]+ – t_R = 7.626 min – $C_{35}H_{36}N_4O_5$ – Pheophorbide a isomer 1 11

Figure S22. Proposed fragmentation pathway for Compound M2385 12

Figure S23. Compound M2789 – *m/z* 593.2763 – [M+H]+ – t_R = 8.064 min – $C_{35}H_{36}N_4O_5$ – Pheophorbide a isomer 2 12

Figure S24. Compound M6441– *m/z* 808.6011 – [M]+ – t_R = 13.721 min – $C_{54}H_{80}O_5$ – Siphonaxanthin ester 14:1 13

Figure S25. Compound M5328 – *m/z* 807.5936 – [M+H]+ – t_R = 11.231 min – $C_{54}H_{80}O_6$ – Hydro-siphonaxanthin ester 14:1 13

Figure S26. Mass spectra and fragmentation pattern of Siphonaxanthin 14

Figure S27. Compound M5763 – *m/z* 803.5596 – [M+Na]+ – t_R = 12.187 min – $C_{52}H_{76}O_5$ – Siphonein 15

Figure S28. Compound M2546 – *m/z* 601.4246 – [M+H]+ – t_R = 7.787 min – $C_{40}H_{56}O_4$ – Prasinoxanthin 15

Figure S29. M1889 – *m/z* 600.4174 – [M]+ – t_R = 7.03 min – $C_{40}H_{56}O_4$ – Violaxanthin 16

Figure S30. Compound M2211 – *m/z* 659.4307 – [M+H]+ – t_R = 7.391 min – $C_{42}H_{58}O_6$ – Fucoxanthin. **A:** MS^2, **B:** MS1 16

Figure S31. Compound M5935 – *m/z* 534.4882 – [M+H]+ – t_R = 12.589 min – $C_{34}H_{63}NO_3$ – Cer(d18:2/16:1) 16

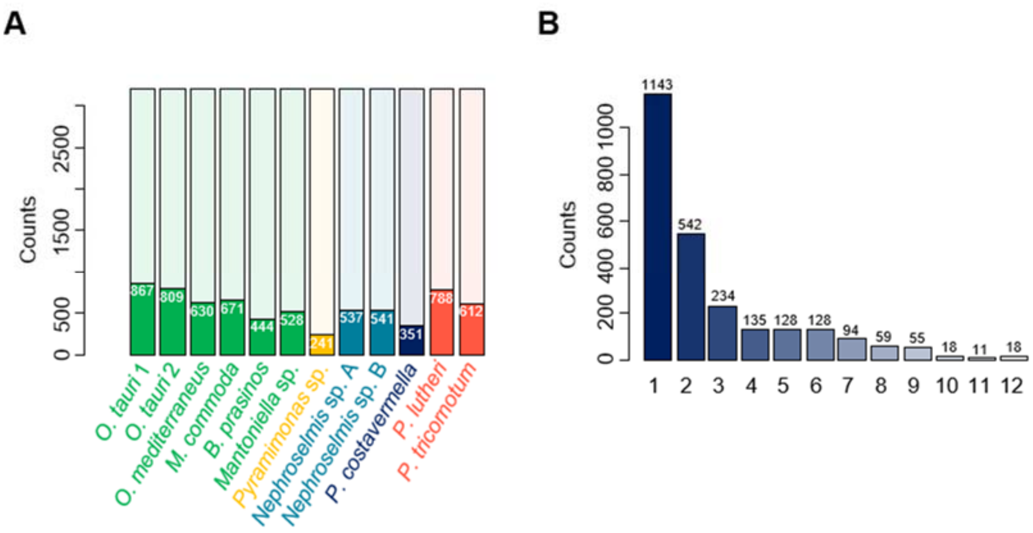

Figure S1. Stacked histogram of detected compounds (bottom bars, intense colors) among total detected compounds (upper bars, blurred colors) in the twelve strains of algae (**A**). Histogram of counts of shared compounds between different strains (abscise axis indicates the number of strains sharing the same compound). Most of compounds (1143) are uniquely detected in one strain (1) while only few compounds (18) are detected in every alga (12) (**B**).

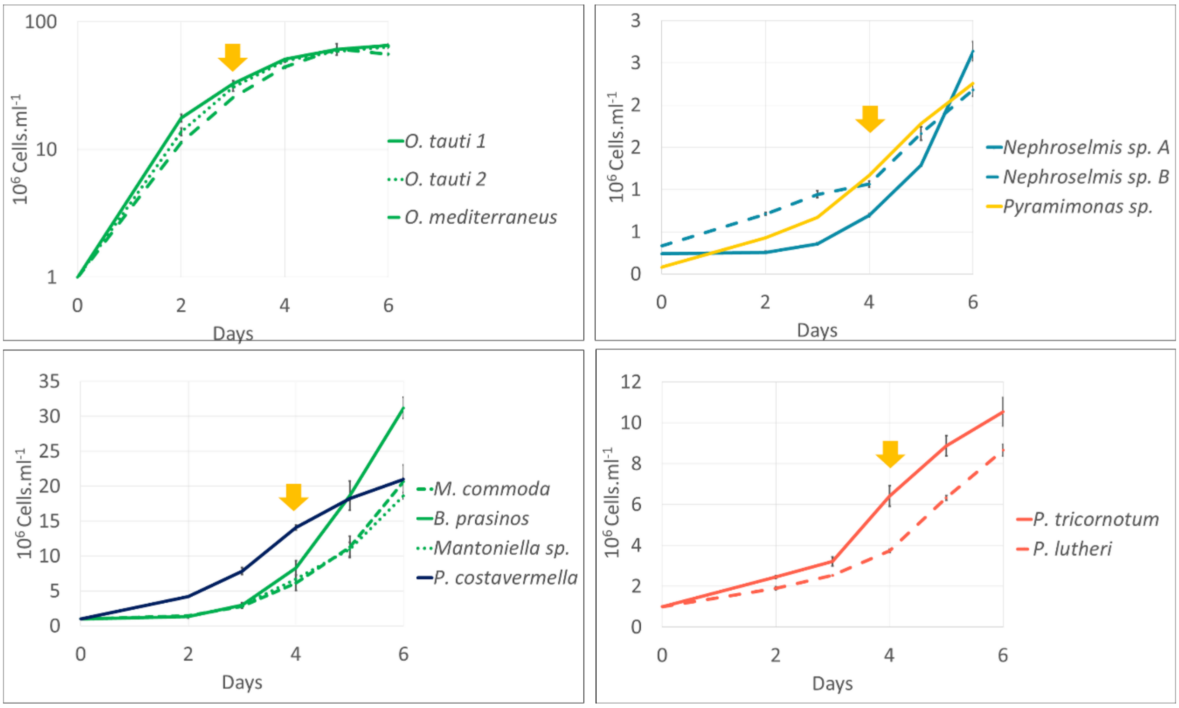

Figure S2. Microalgae concentration growth curves over time. Yellow arrows: metabolite extraction was performed at 3 days post-inoculation for Ostreococcus species and 4 days post inoculation for the others.

Table S1. Algal and bacterial concentration (cells.ml^{-1}) at sampling day for the twelve algae species. RCC for Roscoff Culture Collection numbers for strain identification.

Species	RCC number	Algal cell concentration (Cells.ml^{-1}) mean ± sd	Bacterial cell concentration (Cells.ml^{-1}) mean ± sd	Bacteria proportion (%)	Sampling day post inoculation
O. tauri 1	6850	$32\times10^6 \pm 1.7\times10^6$	$2.6\times10^4 \pm 0.2\times10^4$	0.07	3
O. tauri 2	4221	$30\times10^6 \pm 2.1\times10^6$	$6.1\times10^4 \pm 0.6\times10^4$	0.2	3
O. mediterraneus	2590	$25\times10^6 \pm 0.1\times10^6$	$5.6\times10^4 \pm 0.8\times10^3$	0.2	3
B. prasinos	4222	$8.3\times10^6 \pm 0.9\times10^6$	$5.0\times10^5 \pm 0.7\times10^5$	5.7	4
M. commoda	827	$2.8\times10^6 \pm 0.3\times10^6$	$2.7\times10^6 \pm 0.3\times10^6$	49	4
Mantoniella sp.	6849	$2.9\times10^6 \pm 0.1\times10^6$	$2.5\times10^6 \pm 1.9\times10^6$	46	4
Pyramimonas sp.	6848	$6.7\times10^5 \pm 0.3\times10^5$	$6.2\times10^5 \pm 1.7\times10^5$	48	4
Nephroselmis sp. A	6846	$3.5\times10^5 \pm 0.1\times10^5$	$3.3\times10^6 \pm 0.2\times10^6$	90	4
Nephroselmis sp. B	6847	$9.4\times10^5 \pm 0.4\times10^5$	$8.4\times10^5 \pm 0.4\times10^4$	8.2	4
P. costavermella	4223	$14\times10^6 \pm 0.3\times10^6$	$1.5\times10^6 \pm 1.3\times10^6$	9.7	4
P. lutheri	6852	$2.5\times10^6 \pm 0.3\times10^4$	$3.5\times10^6 \pm 0.2\times10^6$	58	4
P. tricornutum	6851	$3.2\times10^6 \pm 0.2\times10^6$	$0.4\times10^5 \pm 0.9\times10^4$	1.4	4

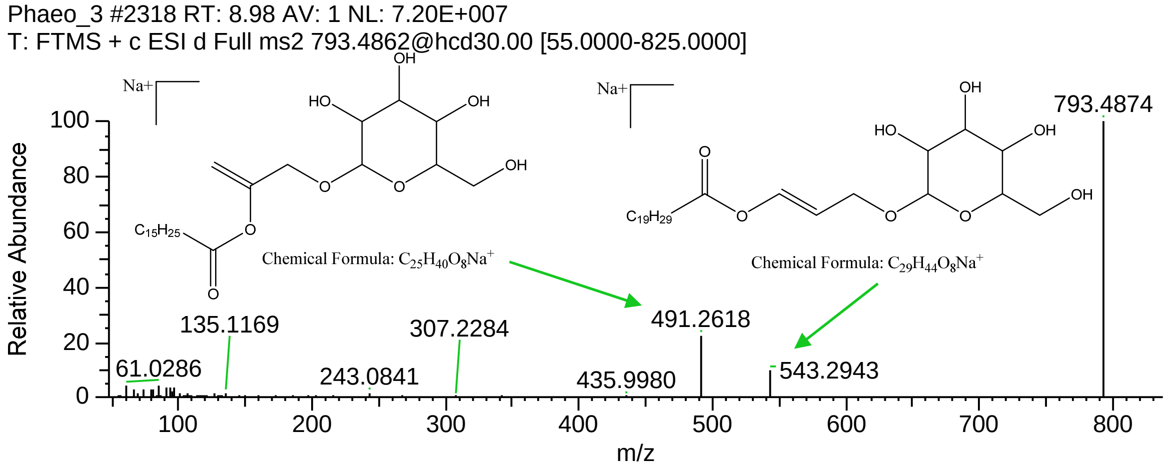

Figure S3. Compound M3629 – *m/z* 793.4874 – [M+Na]$^+$ – t_R = 8.976 min – $C_{45}H_{70}O_{10}$ – MGDG 20:5/16:3

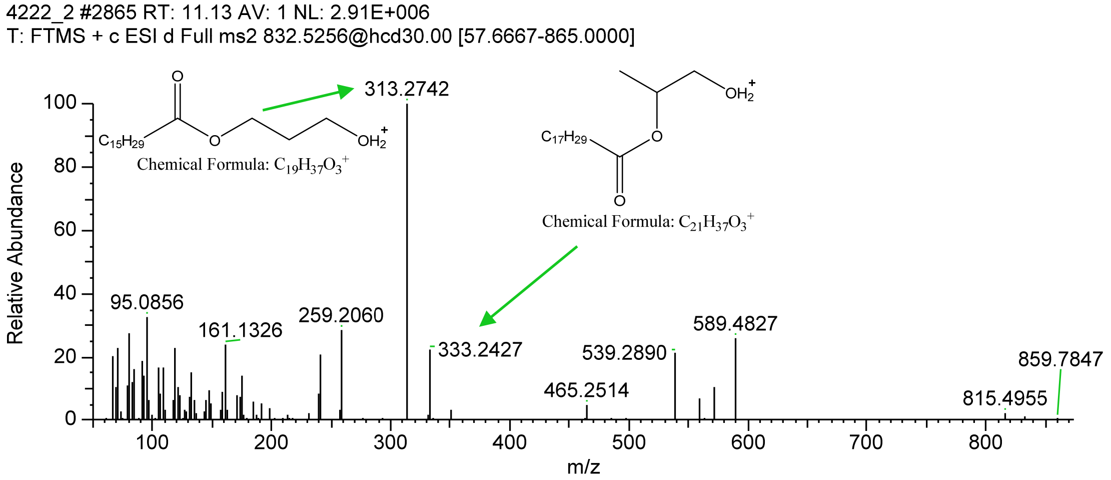

Figure S4. Compound M5273 – *m/z* 815.4985 – [M+H]$^+$ – t_R = 9.167 min – $C_{43}H_{74}SO_{12}$ – SQDG 16:0/18:4

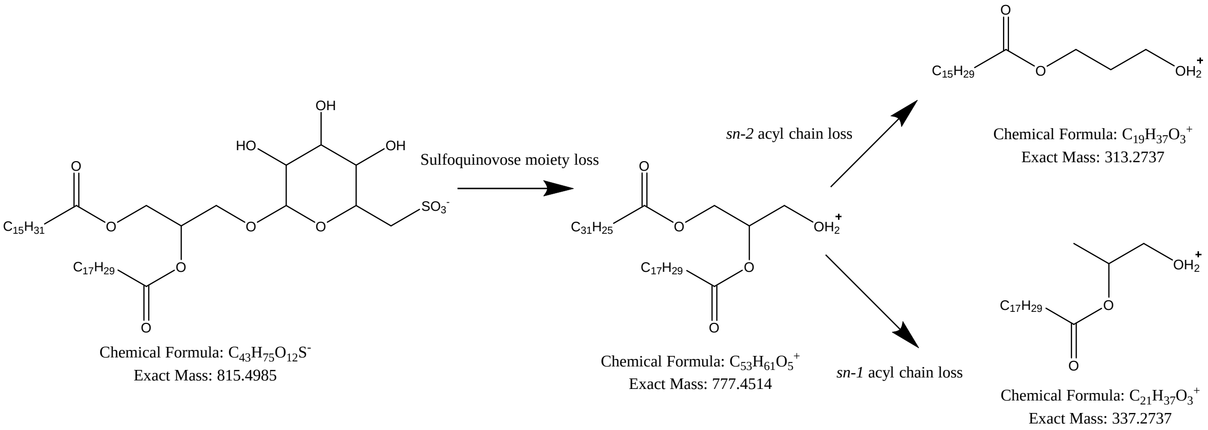

Figure S5. Fragmentation pathway proposed for SQDGs

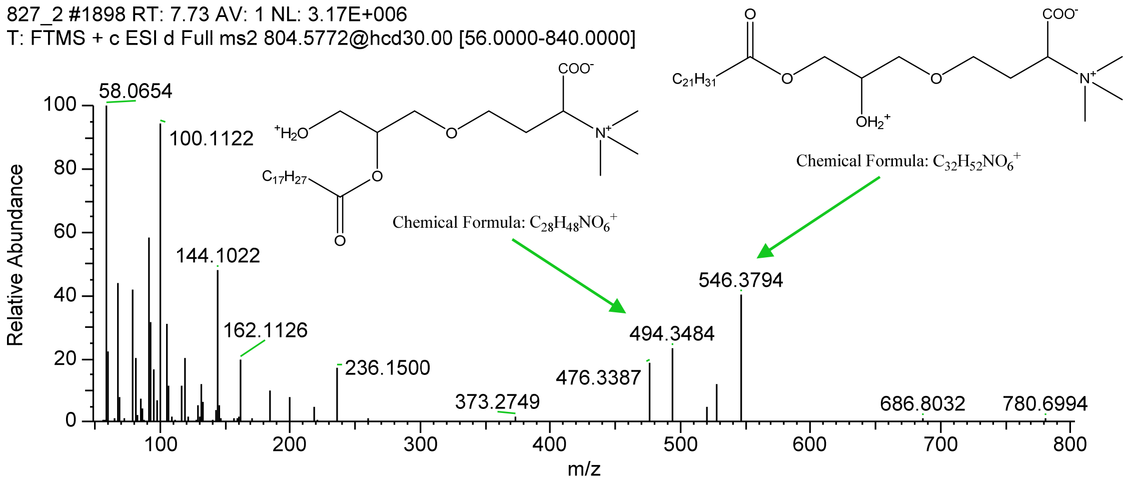

Figure S6. Compound M2531 – *m/z* 804.578 – $[M+H]^+$ – t_R = 8.976 min – $C_{50}H_{77}NO_7$ – DGTS 22:6/18:4

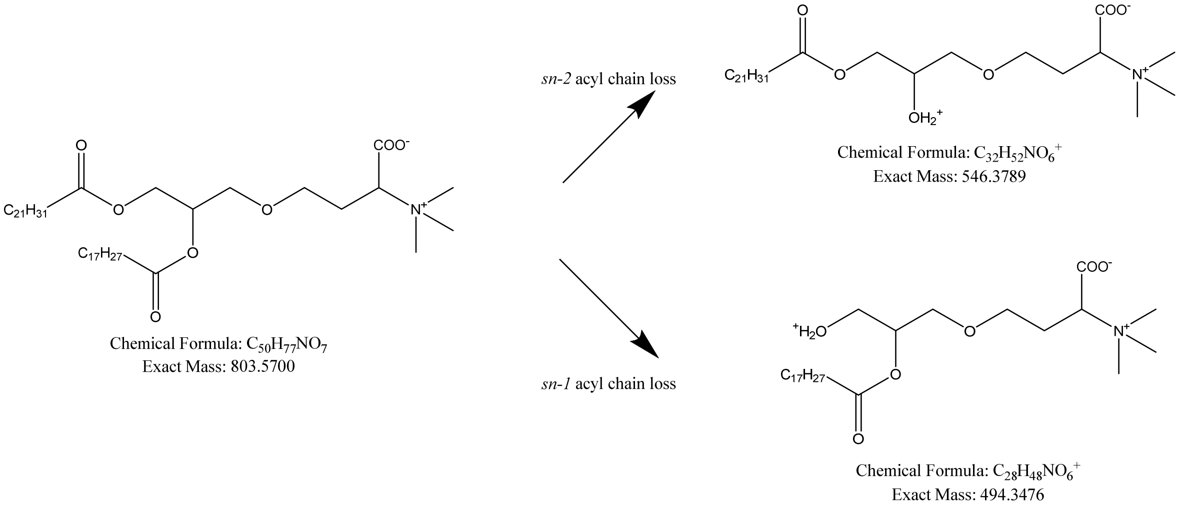

Figure S7. Fragmentation pathway proposed for DGTSs (Fragmentation pattern of the DGTAs are similar)

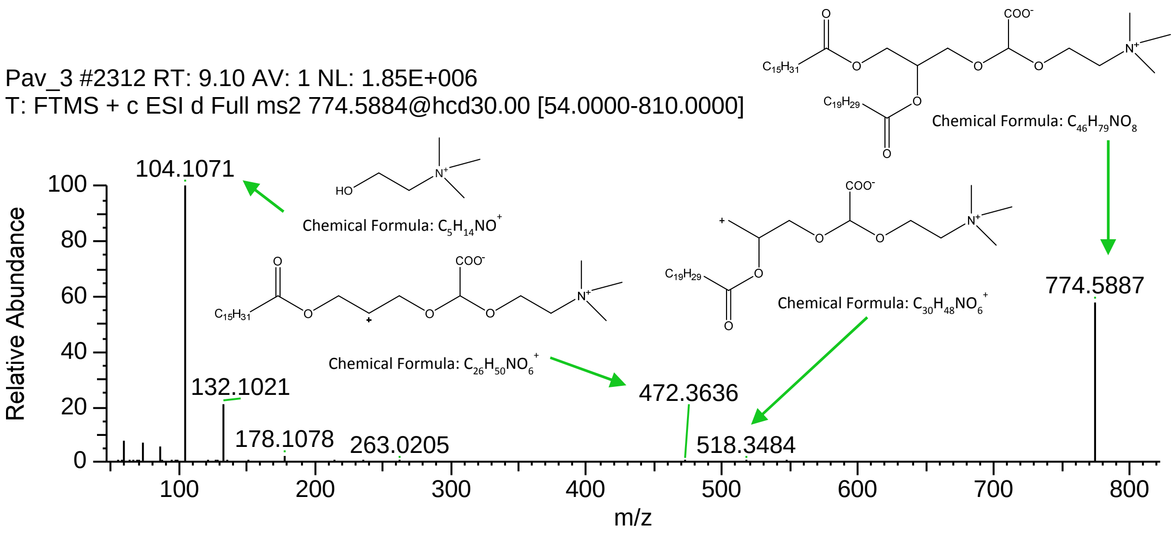

Figure S8. Compound M3834 – *m/z* 774.5888 – $[M+H]^+$ – t_R = 9.167 min – $C_{46}H_{79}NO_8$ – DGCC 16:0/20:5

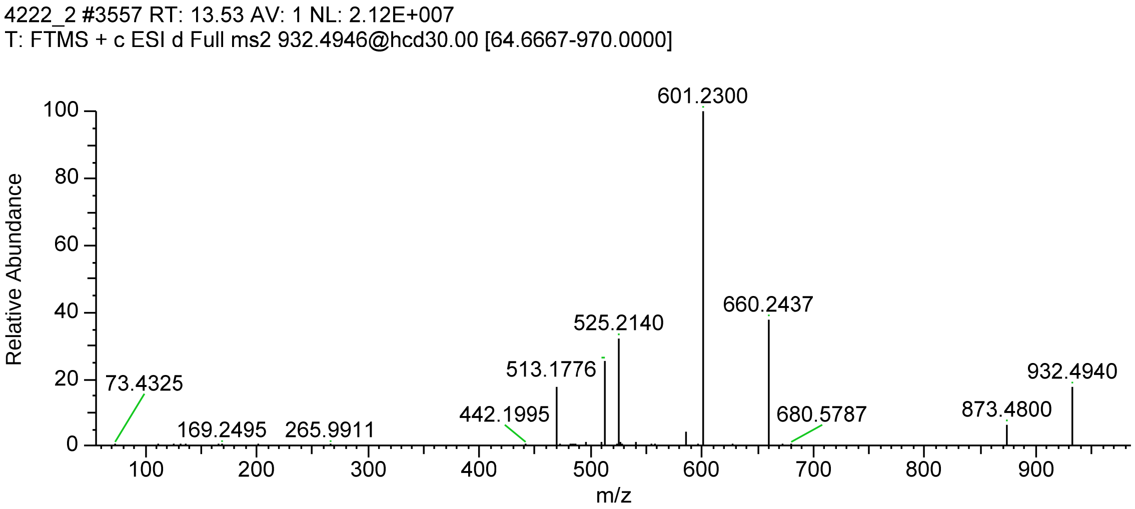

Figure S9. Compound M6284 – *m/z* 932.4940 – $[M]^+$ – t_R = 13.397 min – $C_{56}H_{68}MgN_4O_7$ – Chlorophyll a derivative 1

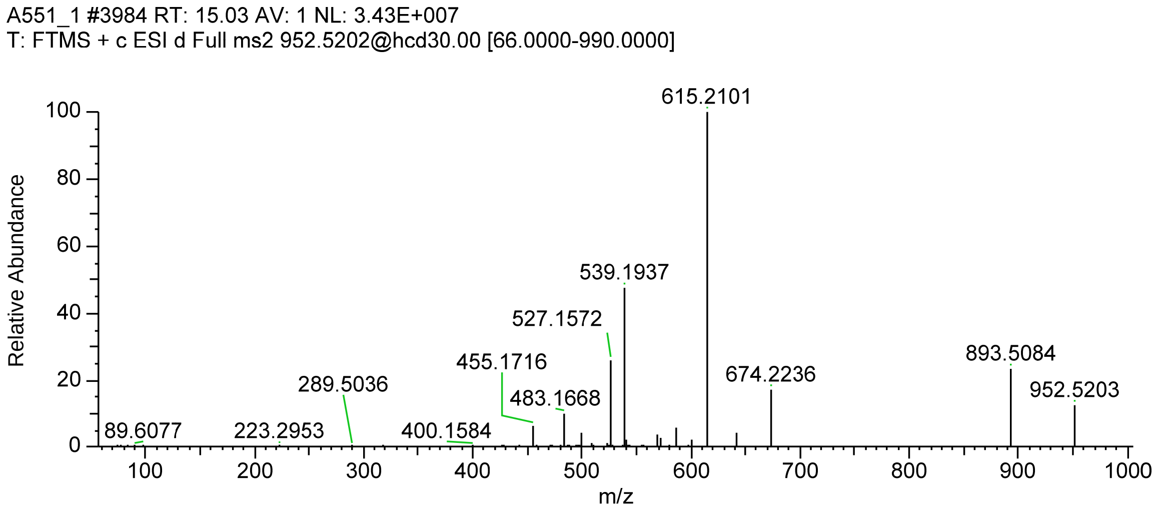

Figure S10. Compound M6982 – *m/z* 952.5200 – [M]$^+$ – t_R = 15.072 min – $C_{56}H_{72}MgN_4O_8$ – Chlorophyll a derivative 2

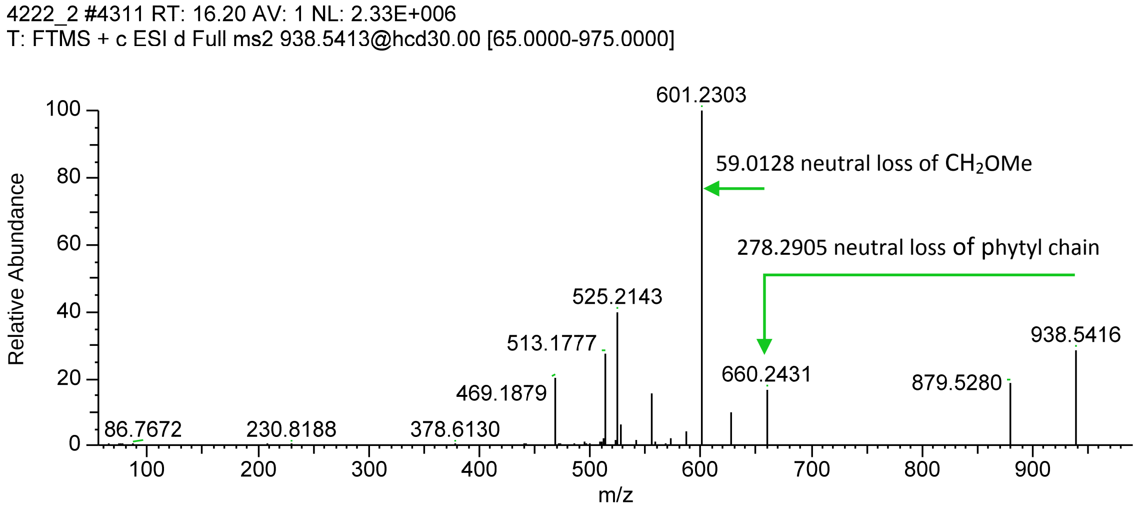

Figure S11. Compound M7441 – *m/z* 938.5412 – [M]$^+$ – t_R = 16.286 min – $C_{56}H_{74}MgN_4O_7$ – Chlorophyll a derivative 3

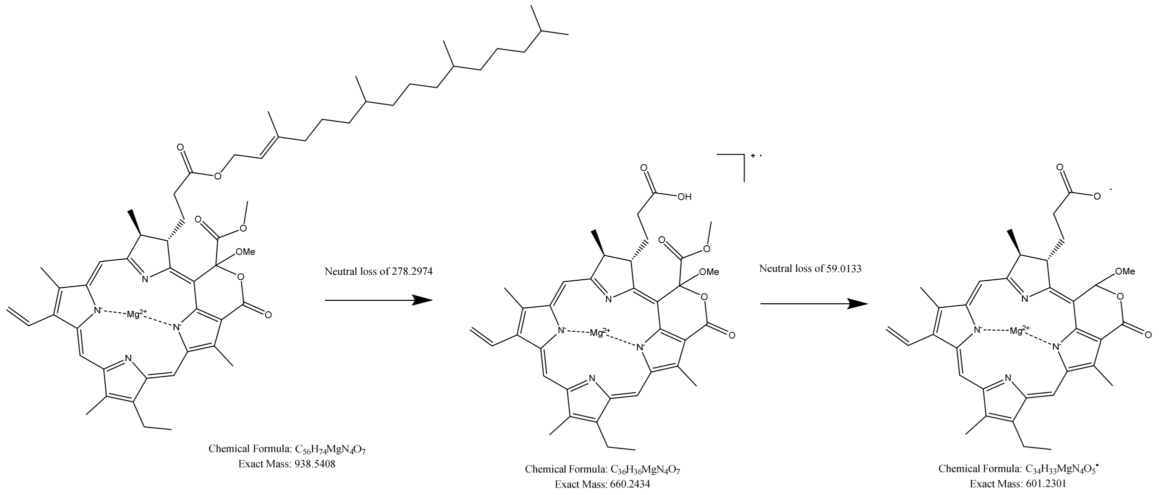

Figure S12. Proposed fragmentation pathway for Compound M7441

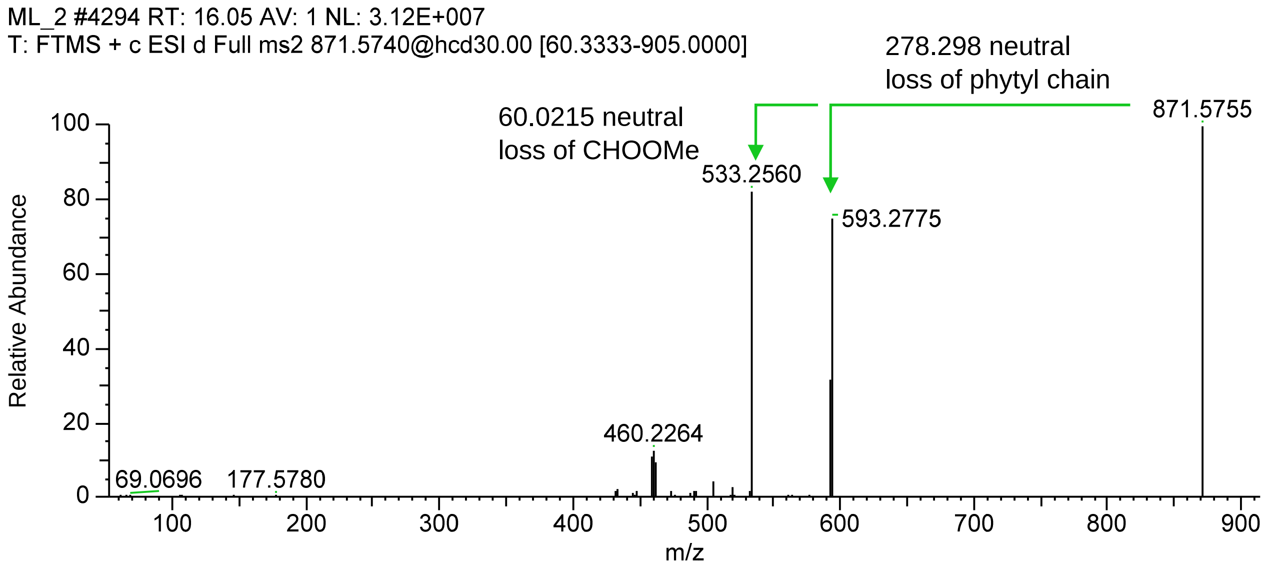

Figure S13. Compound 7354 – *m/z* 871.5740 – [M+H]+ – t_R = 16.096 min – $C_{55}H_{74}N_4O_5$ – Pheophytin a isomer 1

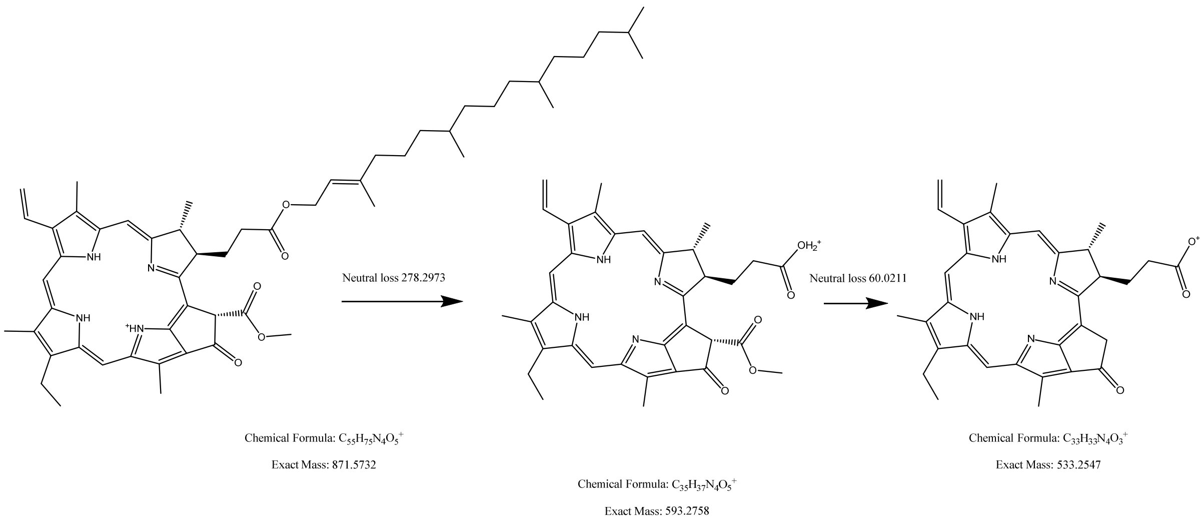

Figure S14. Proposed fragmentation pathway for Compound M7354

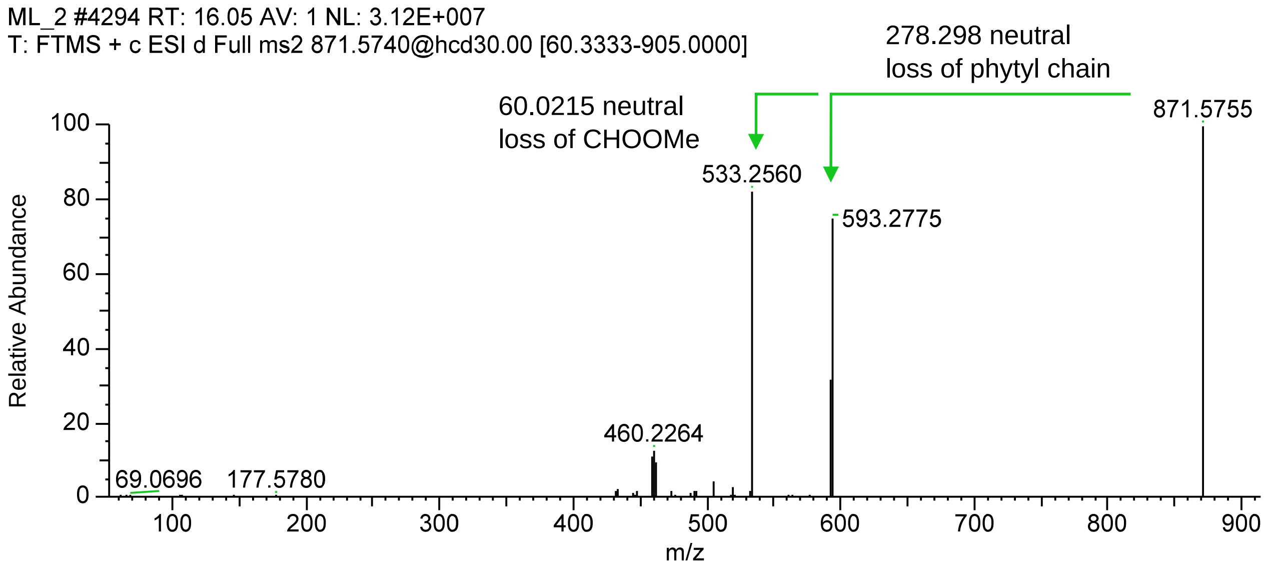

Figure S15. Compound 7377 – *m/z* 871.5740 – [M+H]$^+$ – t_R = 16.169 min – $C_{55}H_{74}N_4O_5$ – Pheophytin a isomer 2

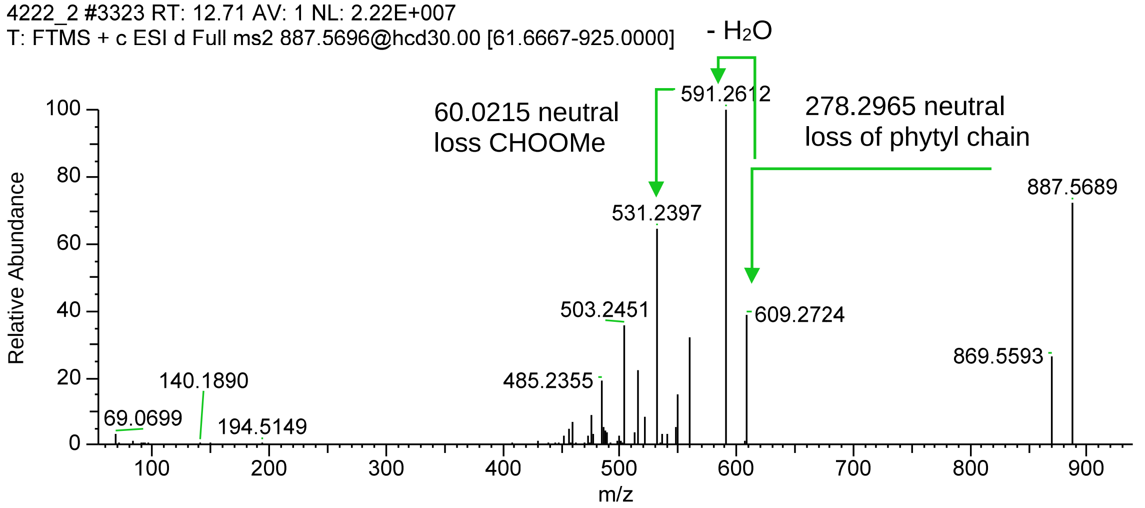

Figure S16. Compound M5963 – *m/z* 887.5688 – [M+H]$^+$ – t_R = 12.658 min – $C_{55}H_{74}N_4O_6$ – Hydroxy-pheophytin isomer 1

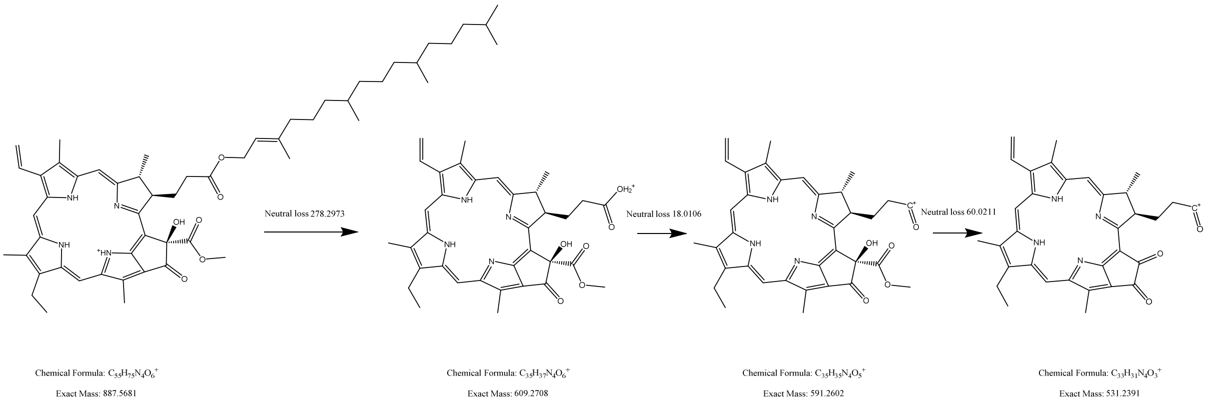

Figure S17. Proposed fragmentation pathway for Compound M5963

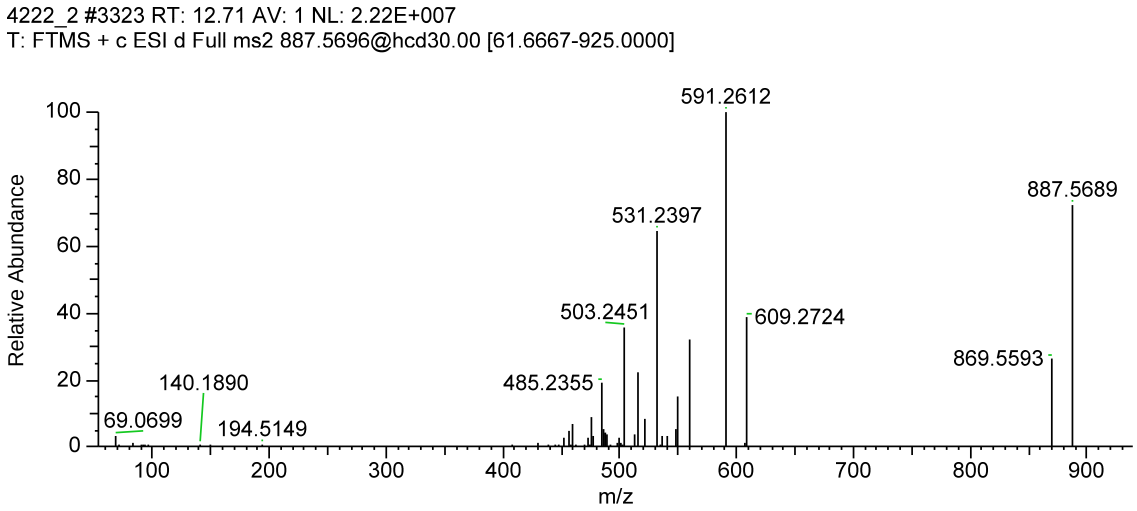

Figure S18. Compound M6021 – *m/z* 887.5688 – $[M+H]^+$ – t_R = 12.779 min – $C_{55}H_{74}N_4O_6$ – Hydroxy-pheophytin isomer 2

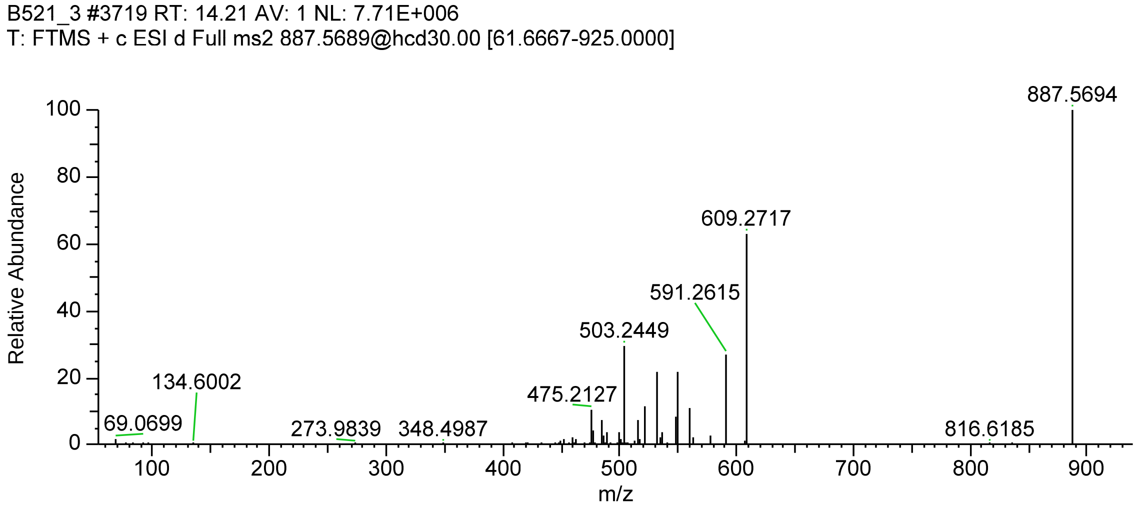

Figure S19. Compound M6695 – *m/z* 887.5688 – $[M+H]^+$ – t_R = 14.279 min – $C_{55}H_{74}N_4O_6$ – Hydroxy-pheophytin isomer 3

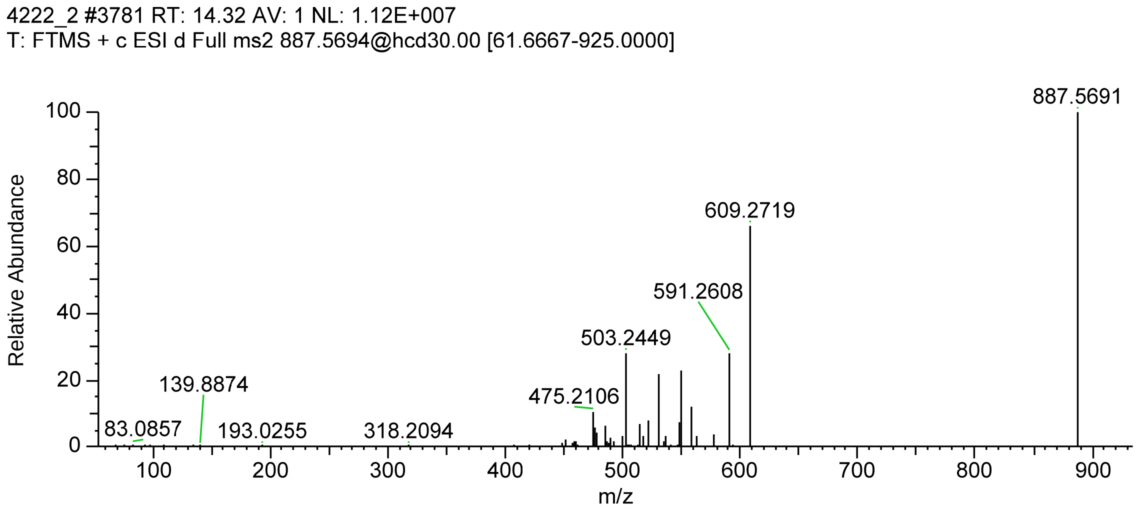

Figure S20. Compound M6726 – *m/z* 887.5688 – $[M+H]^+$ – t_R = 14.396 min – $C_{55}H_{74}N_4O_6$ – Hydroxy-pheophytin isomer 4

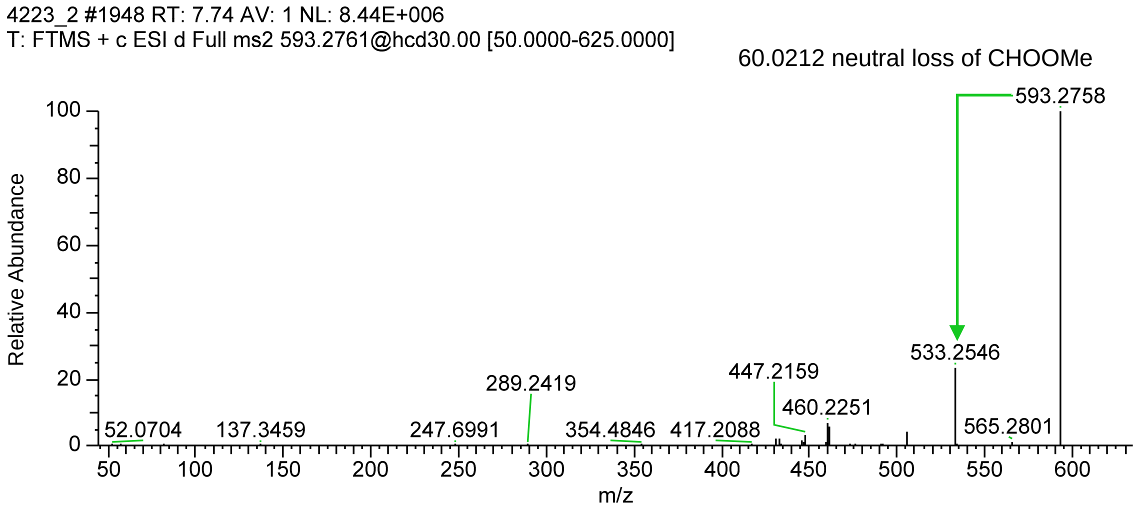

Figure S21. Compound M2385 – *m/z* 593.2763 – $[M+H]^+$ – t_R = 7.626 min – $C_{35}H_{36}N_4O_5$ – Pheophorbide a isomer 1

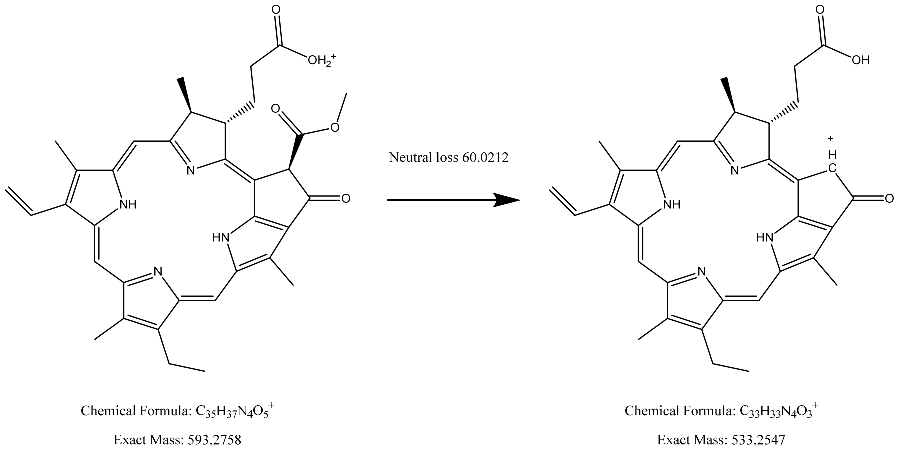

Figure S22. Proposed fragmentation pathway for Compound M2385

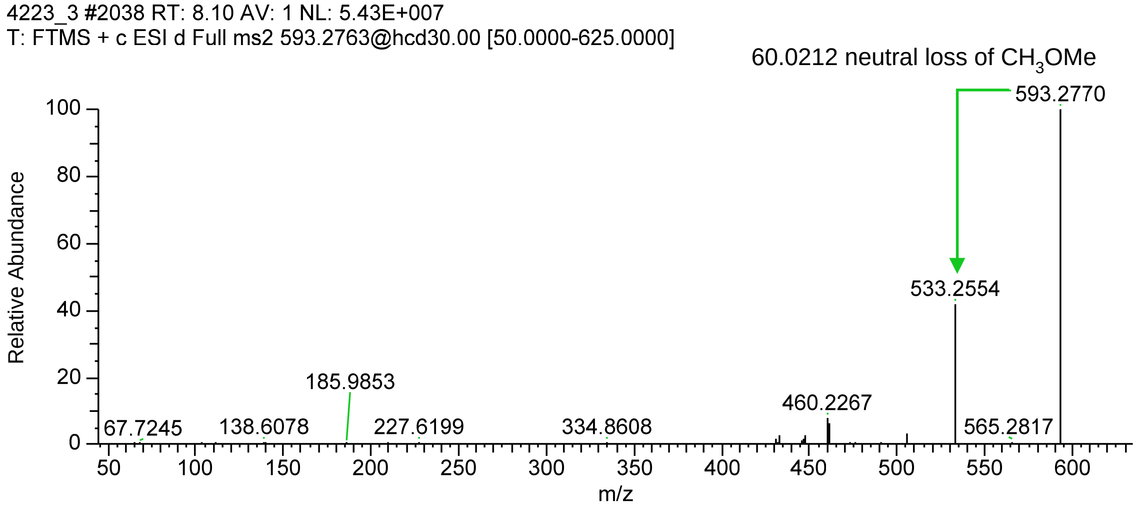

Figure S23. Compound M2789 – *m*/*z* 593.2763 – $[M+H]^+$ – t_R = 8.064 min – $C_{35}H_{36}N_4O_5$ – Pheophorbide a isomer 2

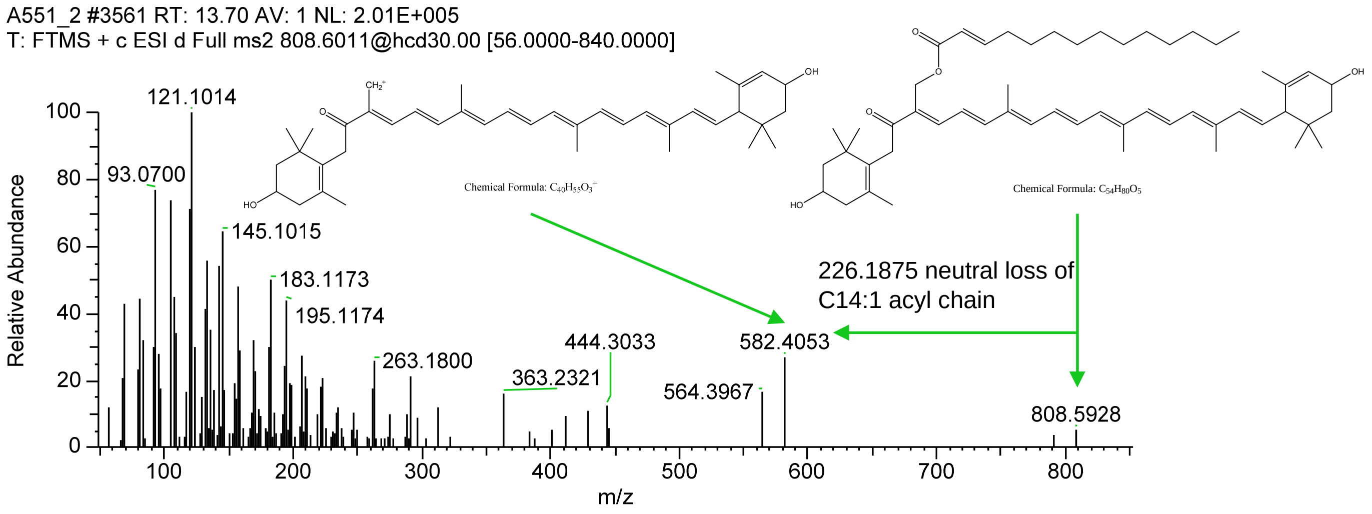

Figure S24. Compound M6441– *m/z* 808.6011 – [M]$^+$ – t_R = 13.721 min – $C_{54}H_{80}O_5$ – Siphonaxanthin ester 14:1

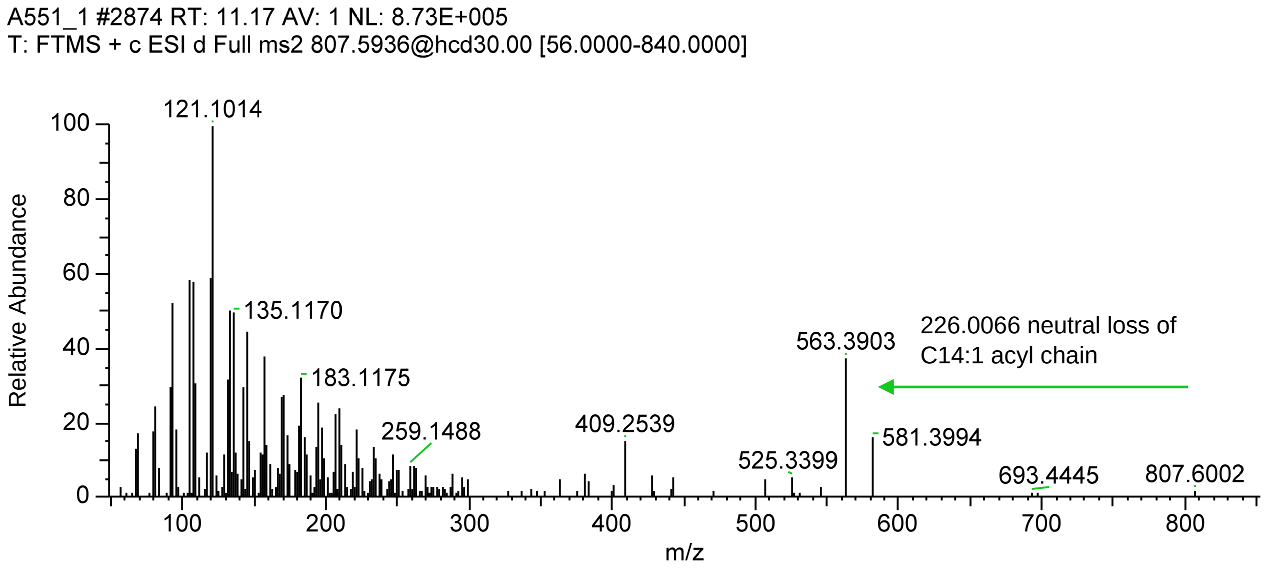

Figure S25. Compound M5328 – *m/z* 807.5936 – [M+H]$^+$ – t_R = 11.231 min – $C_{54}H_{80}O_6$ – Hydro-siphonaxanthin ester 14:1

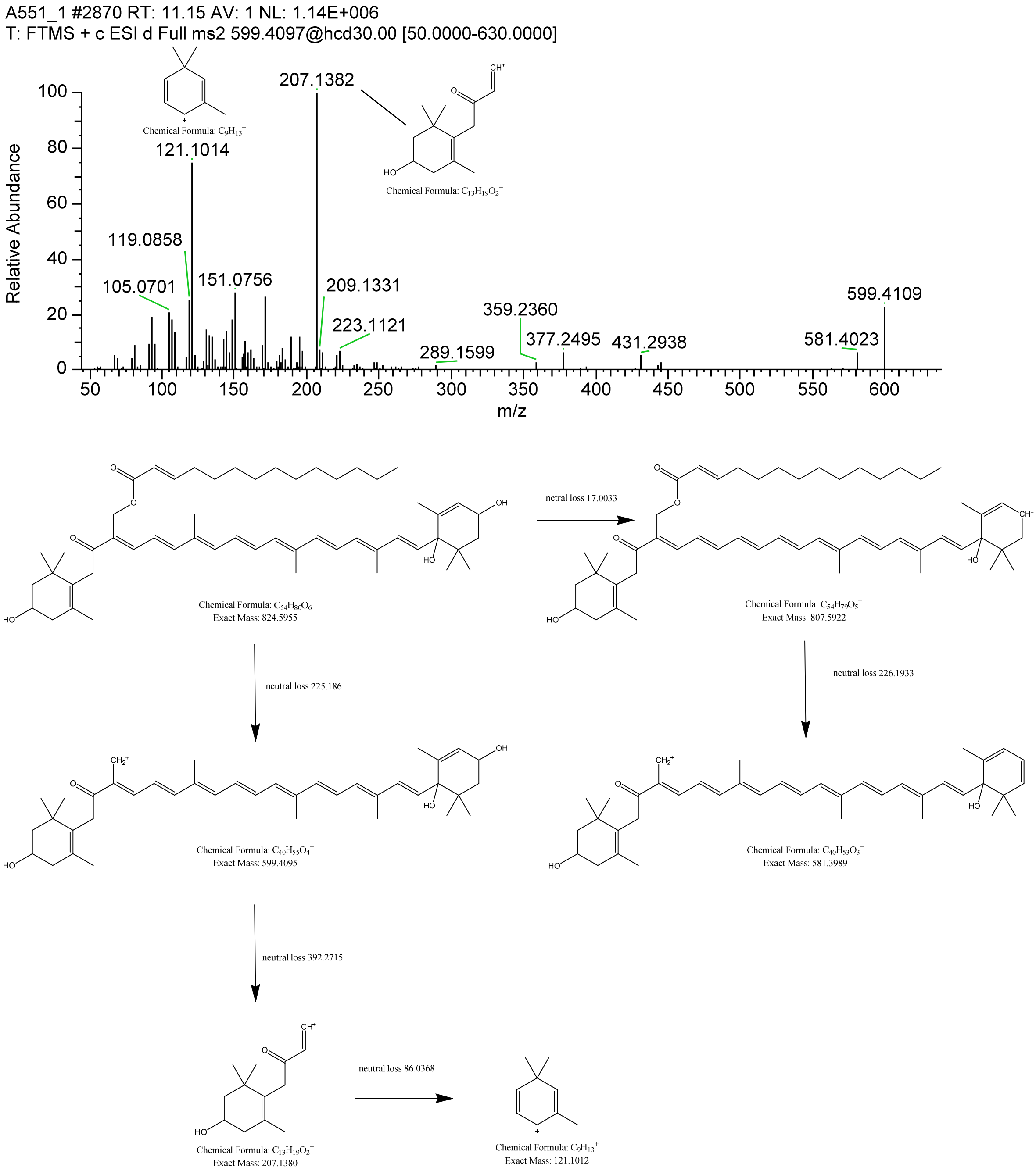

Figure S26. Mass spectra and fragmentation pattern of Siphonaxanthin

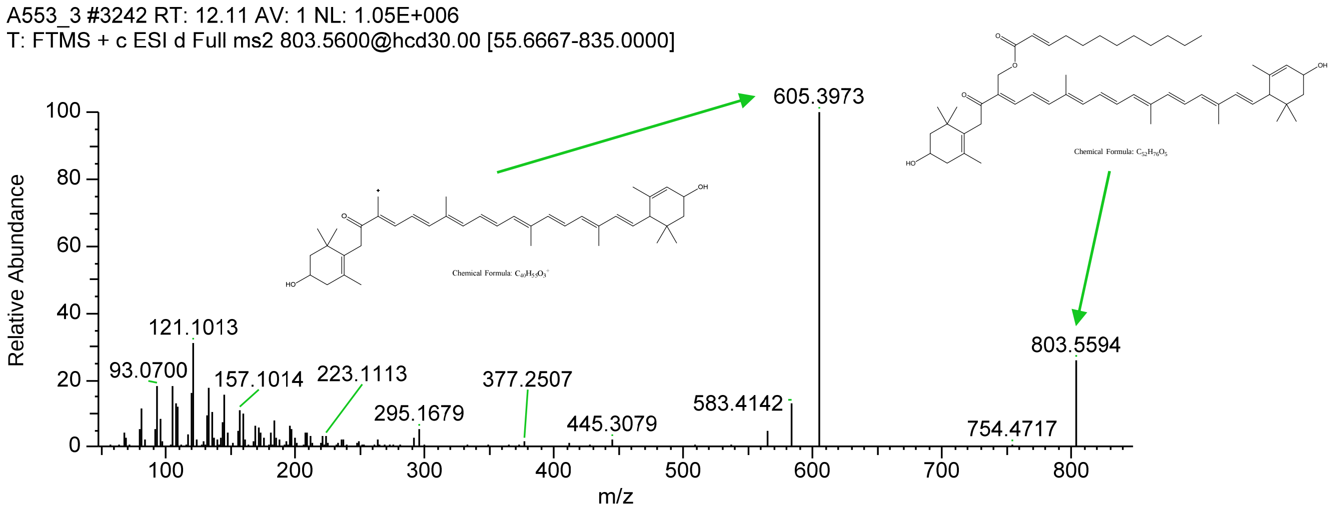

Figure S27. Compound M5763 – *m/z* 803.5596 – [M+Na]$^+$ – t$_R$ = 12.187 min – $C_{52}H_{76}O_5$ – Siphonein

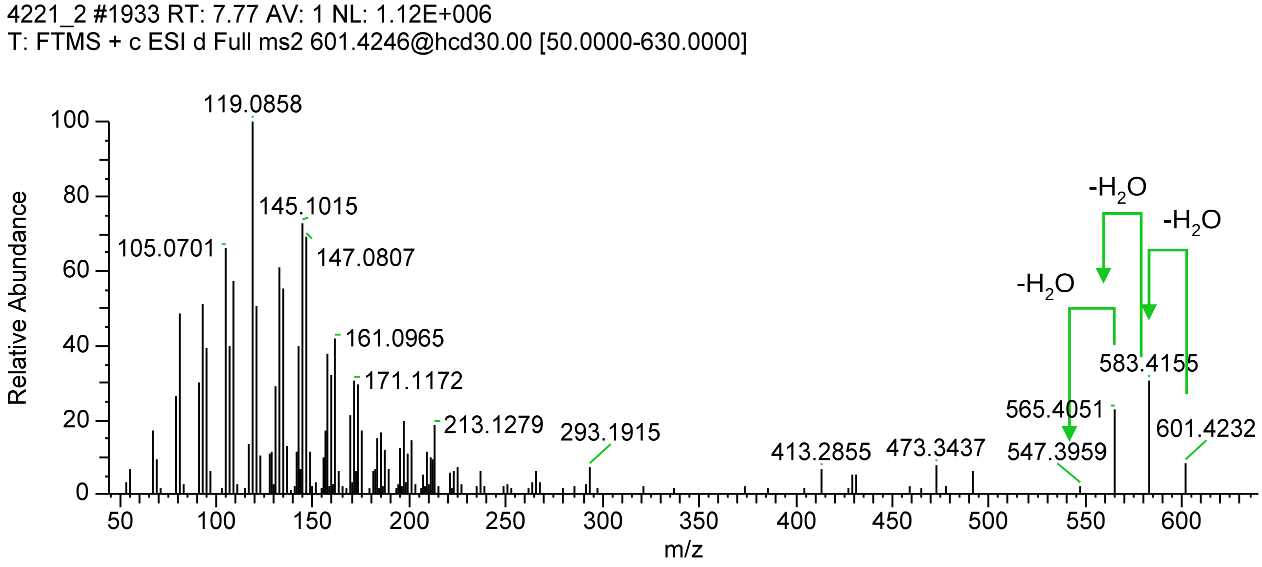

Figure S28. Compound M2546 – *m/z* 601.4246 – [M+H]$^+$ – t$_R$ = 7.787 min – $C_{40}H_{56}O_4$ – Prasinoxanthin

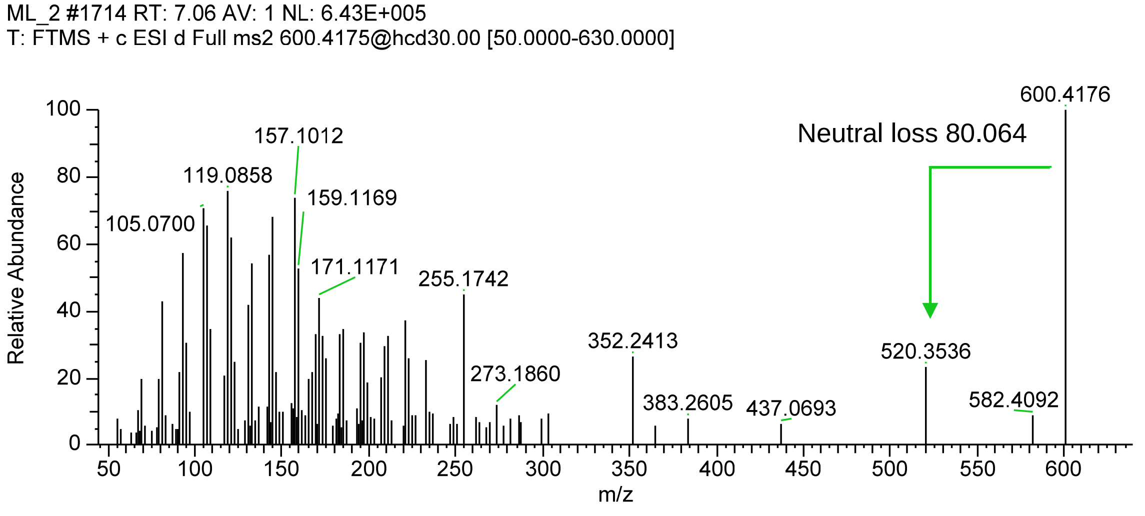

Figure S29. M1889 – *m/z* 600.4174 – [M]$^+$ – t_R = 7.03 min – $C_{40}H_{56}O_4$ – Violaxanthin

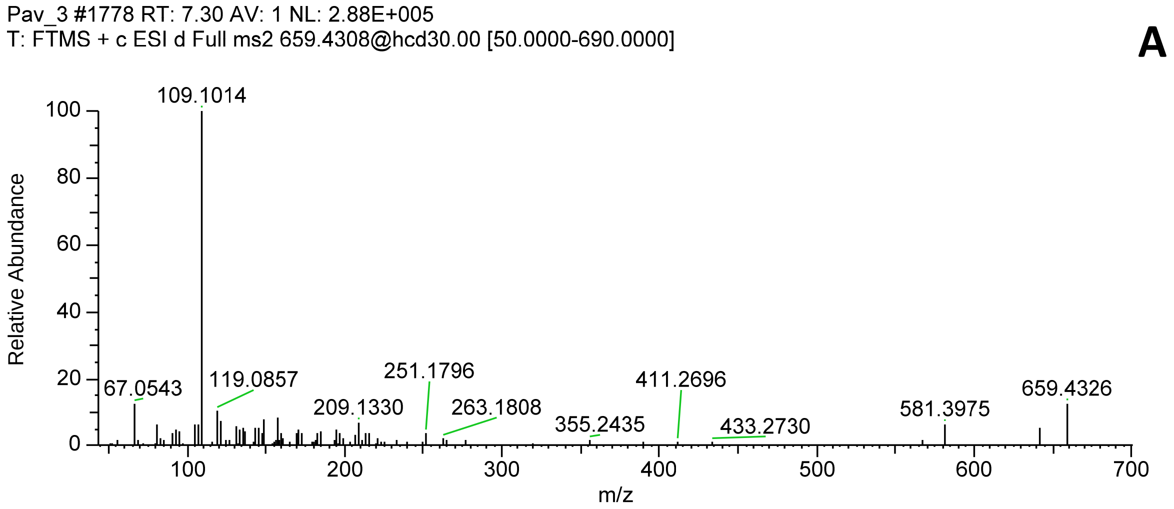

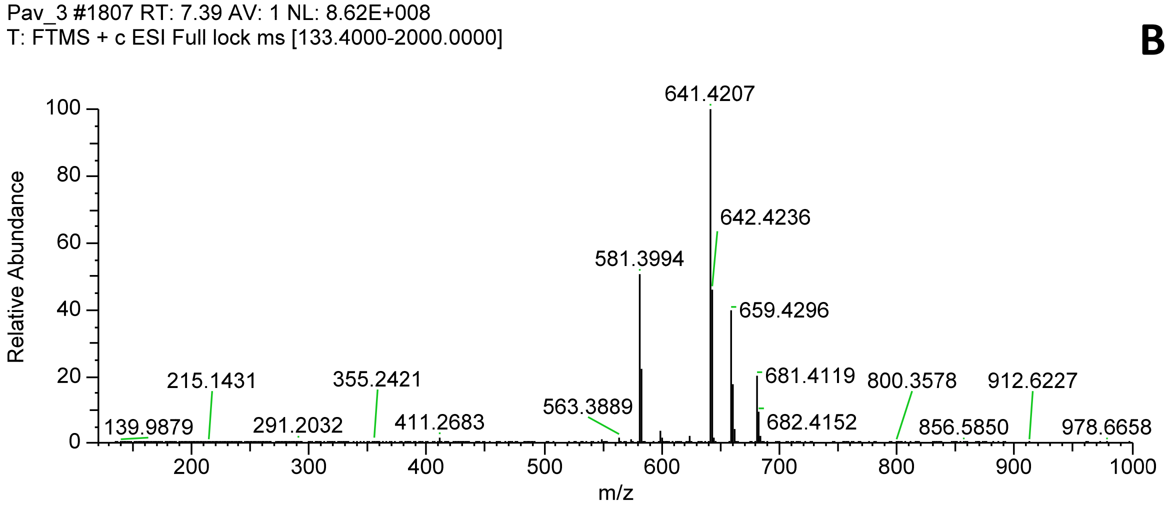

Figure S30. Compound M2211 – *m/z* 659.4307 – [M+H]$^+$ – t_R = 7.391 min – $C_{42}H_{58}O_6$ – Fucoxanthin. **A:** MS^2, **B:** MS1

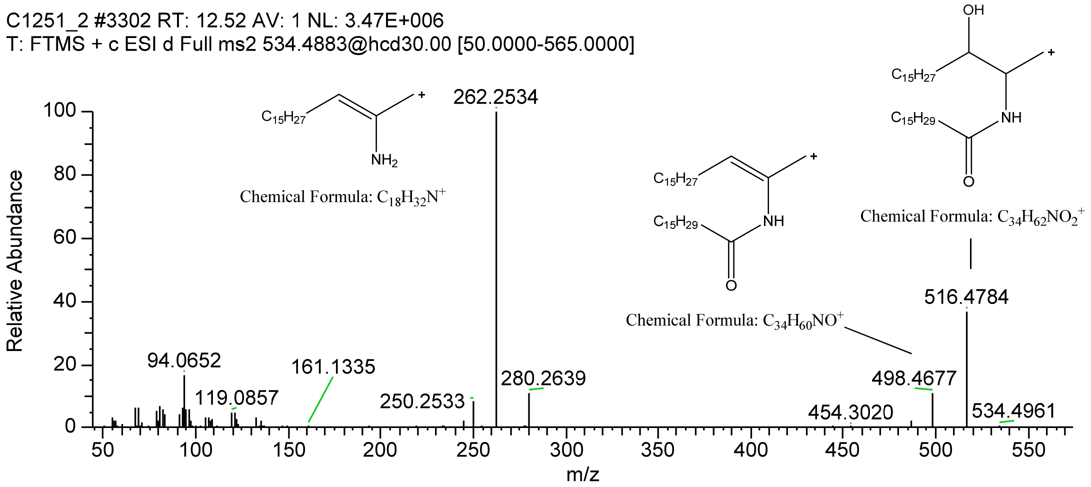

Figure S31. Compound M5935 – *m/z* 534.4882 – [M+H]$^+$ – t_R = 12.589 min – $C_{34}H_{63}NO_3$ – Cer(d18:2/16:1)
